# Language network functional connectivity varies by aphasia type and severity

**DOI:** 10.1016/j.nicl.2026.104030

**Published:** 2026-07-03

**Authors:** Svetlana V. Kuptsova, Arianna N. LaCroix

**Affiliations:** Department of Speech, Language, and Hearing Sciences, Purdue University, West Lafayette, IN 47907, USA

**Keywords:** Aphasia, Functional connectivity, Types of aphasia, Aphasia severity, Resting-state fMRI

## Abstract

Aphasia is increasingly understood as a disorder of disrupted language networks rather than damage to isolated language regions. However, most resting-state functional connectivity studies treat people with aphasia as a single group or classify individuals by overall severity, potentially obscuring qualitative differences in network organization across aphasia types. The present study examined how resting-state functional connectivity varies across aphasia types and how aphasia severity relates to network organization following left-hemisphere stroke. Resting-state fMRI data were analyzed from 89 individuals in the chronic stage of recovery drawn from the open-source Aphasia Recovery Cohort dataset. Network-level and ROI-to-ROI functional connectivity analyses were conducted across 32 predefined dorsal and ventral stream language regions. Lesion-overlap analyses were additionally performed to characterize group-level lesion distributions. Network-level analyses revealed relatively limited effects, whereas ROI-to-ROI analyses demonstrated substantial heterogeneity across aphasia subgroups. Broca's aphasia and severe aphasia demonstrated clearer and more spatially convergent connectivity patterns, whereas anomic, mild, and moderate aphasia groups showed greater heterogeneity and limited group-level effects. Groups showing clearer functional connectivity patterns were also characterized by greater convergence in lesion distributions. Within Broca's aphasia, milder impairment was associated with stronger left-hemisphere and interhemispheric connectivity. Overall, aphasia type-based analyses revealed more differentiated connectivity patterns than severity-based groupings alone. These findings suggest that post-stroke language network organization varies across aphasia types characterized by partially shared clinical and lesion features and may not be fully captured by severity measures alone.

## Highlights

• Aphasia type analyses revealed clearer connectivity patterns than severity alone.
• ROI-to-ROI analyses revealed clearer subgroup patterns than global network analyses.
• Groups with greater lesion and clinical similarity showed clearer FC patterns.

## 1 Introduction

Language processing in the neurotypical brain is supported by a well-established dual-stream architecture (Hickok and Poeppel, 2007). According to this framework, the ventral stream, anchored in the superior and middle temporal lobes, maps speech sounds onto meaning and is organized bilaterally with a left-hemisphere bias. In contrast, the dorsal stream, which includes the posterior planum temporale and posterior frontal regions, transforms acoustic signals into articulatory motor representations and is strongly left lateralized. Because these pathways support distinct components of language processing, damage to either stream commonly results in aphasia, an acquired disorder in which individuals have impairments in expressive and/or receptive language (Dronkers and Ivanova, 2023; Flowers et al., 2016). Importantly, aphasia is increasingly understood as a network disorder, in which language deficits reflect disruption of large-scale functional interactions rather than damage to isolated cortical regions (Baldassarre et al., 2019; Meier, 2022). As a result, post-stroke language outcomes depend not only on lesion location and extent, but also on the integrity and organization of the remaining language network.

Following stroke, intact brain regions can partially assume functions of damaged tissue, and the nature of these network-level changes varies according to lesion location, extent, and network integrity (Brownsett et al., 2014; Geranmayeh et al., 2017; Tsvetkova, 2004). Across numerous studies, activation within preserved left-hemisphere perilesional regions has been most consistently associated with better language outcomes (Fridriksson et al., 2010; LaCroix et al., 2021; Nenert et al., 2018; Price and Crinion, 2005; Warren et al., 2009; Wilson and Schneck, 2020). When left-hemisphere damage is extensive, recruitment of right-hemisphere homologs is frequently observed, although its functional role may vary across individuals and clinical presentations (Blasi et al., 2002; Sebastian and Kiran, 2011; Skipper-Kallal et al., 2017). Together, these findings suggest that successful language function following stroke depends on the coordinated recruitment of distributed language regions, motivating investigation of functional interactions among these regions in addition to regional activation patterns.

Resting-state fMRI (rs-fMRI), which measures intrinsic network organization in the absence of an explicit task, offers a valuable approach for investigating post-stroke changes in network organization, particularly in clinical populations (Tie et al., 2014). Because language recovery likely depends on coordinated interactions among distributed language regions rather than isolated regional activation alone, examination of functional connectivity may provide important insight into post-stroke language network organization. In people with aphasia (PWA), rs-fMRI studies have consistently reported reduced functional connectivity among left-hemisphere language regions relative to neurotypical controls (Harvey et al., 2013; Sandberg, 2017; Sebastian et al., 2016; Zhu et al., 2014). Stronger connectivity within preserved language networks, particularly in the left hemisphere, has been associated with better language performance and greater recovery (Nair et al., 2015; Sebastian et al., 2016; Siegel et al., 2016; Zhu et al., 2014). However, reported patterns of functional connectivity vary widely across studies and individuals, likely reflecting heterogeneity in lesion location, aphasia type, severity, and recovery trajectory (Kiran et al., 2015; Rosen et al., 2000). Yet most functional connectivity studies either treat PWA as a single group or relate connectivity to aphasia severity alone, without accounting for aphasia type (Harvey et al., 2013; Sandberg, 2017; Sebastian et al., 2016). As a result, potentially meaningful subtype-related patterns of network organization may be obscured, as individuals with similar severity levels may differ substantially in lesion location, language profile, and residual network organization. Accordingly, incorporating aphasia type alongside severity may provide additional context for understanding heterogeneity in post-stroke language network connectivity.

Aphasia types represent clinically defined patterns of language impairment that often reflect partially shared lesion distributions and residual network organization. For example, Broca's aphasia is typically characterized by nonfluent, effortful, and agrammatic speech with relatively preserved comprehension for simple structures and is most commonly associated with lesions involving the left inferior frontal gyrus, insula, and surrounding frontal–parietal regions (Sheppard and Sebastian, 2021). In contrast, Wernicke's aphasia is characterized by fluent speech accompanied by significant impairments in comprehension, naming, and repetition and is commonly associated with lesions involving temporal and parietal regions (Sheppard and Sebastian, 2021). Conduction aphasia, in turn, is characterized by relatively fluent speech with prominent repetition deficits and phonological errors and is often associated with damage to left temporoparietal regions (Buchsbaum et al., 2011; Sheppard and Sebastian, 2021). Anomic aphasia is comparatively more heterogeneous, arising from lesions affecting multiple cortical and subcortical areas. Despite variability in lesion location, word-finding difficulties remain the defining feature of anomic aphasia, alongside relatively preserved grammar, comprehension, and repetition (Sheppard and Sebastian, 2021).

The present study examines whether patterns of language network functional connectivity differ across aphasia types and whether aphasia severity is associated with variability in these connectivity patterns. To address these questions, we first examined network-level functional connectivity across aphasia types (anomic, conduction, Broca's) and aphasia severities (no aphasia, mild, moderate, severe) within the dorsal stream, ventral stream, and whole language network (dorsal + ventral streams). We then conducted ROI-to-ROI analyses to characterize connection-level functional connectivity differences between groups and patterns of connectivity within groups. Within-group analyses additionally examined associations between aphasia severity and ROI-to-ROI connectivity within each aphasia type. Finally, individual variability and lesion overlap analyses were conducted to contextualize heterogeneity in functional connectivity organization across aphasia types and severities. We hypothesized that functional connectivity patterns would differ across aphasia types, reflecting differences in network organization associated with each aphasia profile, and that aphasia severity would be associated with graded differences in functional connectivity, such that individuals with less severe aphasia would show stronger connectivity within left-hemisphere language regions, whereas individuals with more severe aphasia would show reduced left-hemisphere connectivity and stronger right-hemisphere connectivity.

## 2 Methods and materials

### 2.1 Participants

Data for this study were obtained from The Aphasia Recovery Cohort (ARC) Dataset (Gibson et al., 2024), an open-source repository of chronic post-stroke aphasia that includes resting-state fMRI, structural imaging, and demographic information, and aphasia type and severity measures. The dataset was acquired across multiple studies and acquisition protocols, resulting in substantial heterogeneity in imaging parameters. Because such heterogeneity can introduce scanner- and sequence-related variability in functional connectivity estimates, participant selection was restricted to internally homogeneous acquisition groups.

Based on the available demographic and clinical information, we selected resting-state fMRI data sessions with similar scanning parameters that were collected on the same day as the Western Aphasia Battery (WAB), or as close as possible, ensuring that the interval did not exceed 180 days (*M =* 7.88, *SD* = 19.60). Participants were included if they had a left hemisphere stroke regardless of aphasia presence or diagnosis and were in the chronic stage of stroke recovery (≥ 6 months post-onset). The initial cohort consisted of 103 participants. Three participants were excluded due to structural and functional data being collected in different sessions, and 11 were excluded because of excessive head motion (>3.0 mm). The final group included 89 participants.

**Fig. 1:**
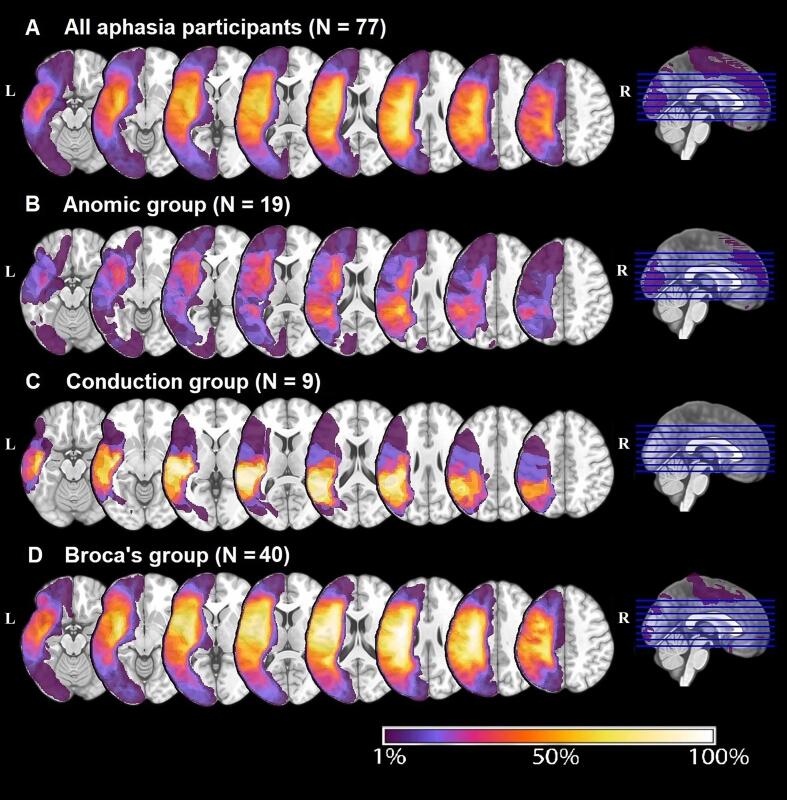
**Lesion overlap maps by aphasia type,** showing the distribution of lesions for all participants with aphasia (A), participants with anomic aphasia (B), conduction aphasia (C), and Broca's aphasia (D). L – left hemisphere; R – right hemisphere.

**Fig. 2:**
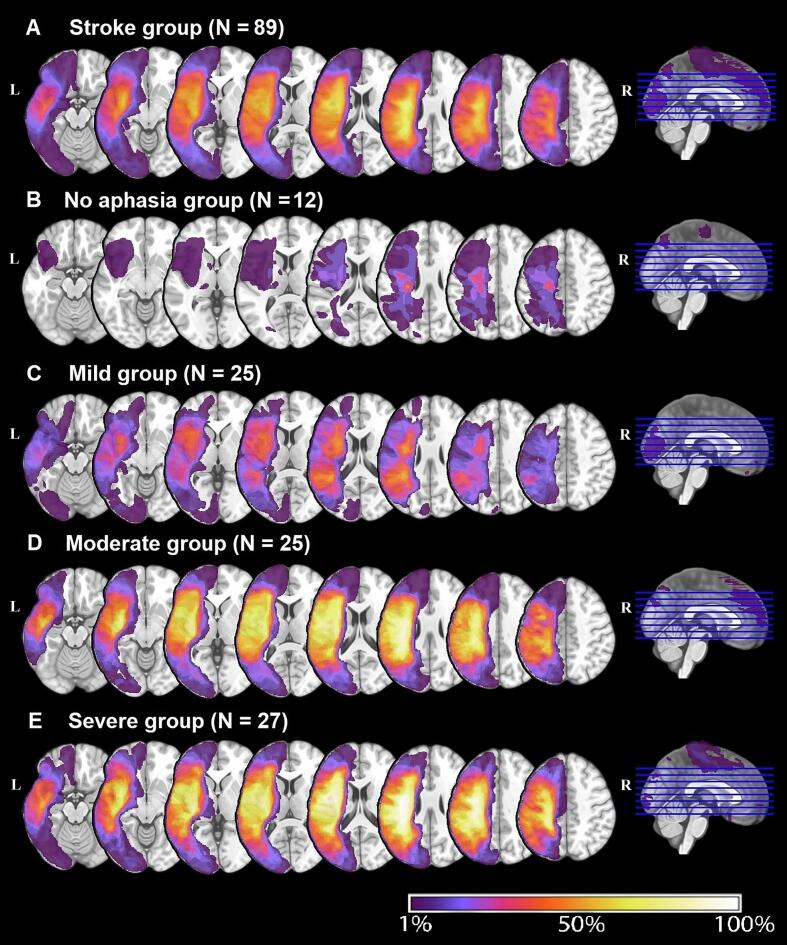
**Lesion overlap maps by aphasia severity,** showing the distribution of lesions for all stroke participants (A), participants with no aphasia (B), mild aphasia (C), moderate aphasia (D), and severe aphasia (E). L – left hemisphere; R – right hemisphere.

The WAB was used to group participants by aphasia type (anomic, Broca's, conduction), which broadly reflects differences in language phenotype and partially shared lesion distributions, and severity, which is a more global index of language impairment and lesion extent. PWA were classified as severe (AQ = 0–50), moderate (AQ = 51–75), or mild (AQ = 76–93.8), with scores ≥93.8 indicating performance within normal limits, which we refer to as the “no aphasia” group. Lesion overlap maps are presented in Fig. 1, Fig. 2 for aphasia type and severity, respectively.

Demographic data for each aphasia type and severity are presented in Table 1, and demographic information for each participant is provided in Supplementary Table A.1. There were no significant differences in age across type (*H*(6) = 2.81, *p* = .832) or severity groups (*H*(3) = 2.70, *p* = .439). In contrast, significant differences in sex distribution were observed across type (*χ*^*2*^(5) = 14.78, *p* = .011) and severity (*χ*^*2*^(3) = 11.49, *p* = .009). Post-hoc Fisher's exact tests revealed significant differences in sex distribution between the Broca's and no aphasia groups (Holm-corrected *p* = .012) and between the severe aphasia and no aphasia groups (Holm-corrected *p* = .008), with a higher proportion of male participants in the Broca's and severe aphasia groups. We also examined whether time between stroke onset and rs-fMRI acquisition differed across groups, and no significant group differences were observed for type (*H*(6) = 4.74, *p* = .578) or aphasia severity (*H*(3) = 2.36, *p* = .502). No significant differences were observed in the number of days between the WAB and rs-fMRI sessions across severity groups (*H*(3) = 6.49, *p* = .089), whereas a significant omnibus effect was observed across types (*H*(6) = 13.86, *p* = .031), although no post-hoc comparisons survived correction for multiple comparisons. We also compared participant-level maximum absolute displacement across groups to ensure that macro-level network comparisons were not artifactually driven by differential in-scanner head movement, a common confounding factor when evaluating stroke populations with varying degrees of motor and clinical impairment (Power et al., 2012; Satterthwaite et al., 2012); no significant group differences were observed for type (*H*(6) = 10.64, *p* = .100) or severity (*H*(3) = 5.26, *p* = .153).

**Table 1:** Demographic data for each group by aphasia type and severity.

Groups	Number of participants (number of males)	Age (years) M ± SD (min-max)	Stroke time post-onset (days) M ± SD (min-max)	WAB score M ± SD (min-max)	Lesion volumes M ± SD (min-max)
**Aphasia Type**					
Anomic	19 (12)	58.32 ± 13.46 (37–78)	1329.42 ± 1477.28 (272–5432)	86.01 ± 6.27 (72.9–92.6)	84,231.21 ± 66,105.19 (15943–304,821)
Broca's	40 (32)	55.77 ± 11.48 (31–75)	1544.12 ± 1711.75 (207–6526)	44.52 ± 18.59 (14.8–77.4)	195,895.5 ± 85,960.41 (80351–411,011)
Conduction	9 (4)	57.78 ± 13.07 (27–67)	1118.00 ± 634.73 (359–2530)	67.29 ± 15.06 (39.8–87.1)	108,710.4 ± 64,317.9 (19049–251,492)
Global	5 (3)	54.40 ± 9.13 (46–65)	1064.80 ± 843.01 (456–2515)	22.92 ± 6.25 (14.5–31.3)	205,602.8 ± 91,982.62 (93788–317,611)
Transcortical Motor	3 (1)	62.67 ± 11.72 (54–76)	548.00 ± 262.02 (371–849)	77.13 ± 0.97 (76.3–78.2)	56,956.67 ± 48,098.6 (14143–109,002)
Wernicke	1 (1)	56.00 ± NA (56–56)	311.00 ± NA (311−311)	69.20 ± NA (69.2–69.2)	176,278 ± NA (176278–176,278)
All Aphasia	77 (53)	56.82 ± 11.81 (27–78)	1355.39 ± 1474.22 (207–6526)	57.61 ± 24.73 (14.5–92.6)	153,113.9 ± 93,299.11 (14143–411,011)
**Aphasia Severity**					
No aphasia	12 (3)	52.58 ± 14.55 (29–72)	1882.25 ± 1655.57 (240–4825)	97.58 ± 1.95 (94.0–99.6)	38,203 ± 45,885.53 (200–128,141)
Mild	25 (16)	57.32 ± 13.99 (27–78)	1170.80 ± 1324.29 (272–5432)	84.89 ± 6.01 (76.3–92.6)	80,502.6 ± 52,815.82 (14143–251,492)
Moderate	25 (15)	54.44 ± 10.38 (31–71)	1491.00 ± 1473.39 (262–6327)	62.03 ± 6.88 (52.1–73.9)	170,220.1 ± 84,114.3 (19049–409,927)
Severe	27 (22)	58.56 ± 10.90 (40–75)	1400.74 ± 1636.30 (207–6526)	28.25 ± 8.97 (14.5–44.5)	204,507.4 ± 90,800.92 (93788–411,011)
All Stroke	89 (56)	56.25 ± 12.21 (27–78)	1426.427 ± 1500.774 (207–6526)	63.00 ± 26.78 (14.5–99.6)	137,620.3 ± 96,637.14 (200–411,011)

Notes: WAB – Western Aphasia Battery; PWA – participants with aphasia; NA – not applicable.

### 2.2 MRI acquisition

All structural and functional data were acquired on Siemens 3 T MRI scanners. For the no aphasia group, data were acquired as follows: EPI (TR = 1850 ms, TE = 30 ms, 34 slices, slice thickness = 3 mm, voxel size = 3.25 × 3.25 × 3.6 mm, and 196 total scans), T1 (TR = 2250 ms, TE = 4.15 ms, 192 acquired slices, reconstructed to 256 sagittal slices, slice thickness = 1 mm, voxel size = 1 × 1 × 1 mm). For the aphasia groups, data were acquired as follows: EPI (TR = 1650 ms, TE = 35 ms, 50 slices, slice thickness = 2 mm, voxel size = 2.4 × 2.4 × 2.4 mm, and 427 total scans), T1 (TR = 2250 ms, TE = 4.11 ms, 192 slices, reconstructed to 256 sagittal slices, slice thickness = 1 mm, voxel size = 1 × 1 × 1 mm).

### 2.3 Lesion reconstruction

Lesion masks provided with the dataset were visually inspected and manually corrected, when necessary, to correspond to the T1 image closest in time to the WAB assessment used in the present analysis. Corrections were performed in ITK-SNAP (Yushkevich et al., 2006; Version 3.4.0-rc1, www.itksnap.org). Original lesion masks were reviewed in native space on the T1 image and subsequently refined using T2 and, when available, FLAIR images to improve delineation of chronic tissue changes, including gliosis, cystic regions, and adjacent white matter degeneration. Corrections were limited to regions where discrepancies between the original lesion mask and visible lesion boundaries were identified; whole-lesion redrawing was not routinely performed. Lesion masks were then normalized to the MNI152 template using the Clinical toolbox for SPM12 (Rorden et al., 2012). Normalized lesion masks were visually inspected in MNI space and manually corrected, when necessary, such as when lesion boundaries extended into the ventricles or beyond the meninges. Lesion overlap maps were generated using the Automated Lesion Identification (ALI) toolbox for SPM12 (Seghier et al., 2008).

### 2.4 Rs-fMRI Preprocessing

Structural MRI and fMRI data were preprocessed in SPM12 (http:// www.fil.ion.ucl.ac.uk/spm). The first 10 volumes of each fMRI session were discarded to allow for signal equilibration. Structural and functional images were manually reoriented to the AC-PC plane. Functional images were realigned, coregistered to the structural image, and segmented. To optimize spatial normalization in the presence of focal lesions, structural images were normalized to the MNI152 template using the Clinical Toolbox for SPM12 (Rorden et al., 2012), with lesion maps incorporated during normalization. The resulting deformation fields were subsequently applied to functional images, which were then smoothed using a 6x6x7 mm FWHM Gaussian kernel. Following normalization, structural and functional images were visually inspected in MNI space to ensure the absence of non-physiological spatial warping or distortion near lesion boundaries. Head motion was assessed using SPM12 realignment parameters. Translational (X, Y, Z) and rotational (pitch, roll, yaw) displacements were visually inspected for each participant. Participants were excluded if the maximum absolute displacement exceeded 3.0 mm and 3° in any direction.

Denoising was performed using CONN's implementation of the CompCor strategy (CONN Toolbox, version 18b; Nieto-Castanon and Whitfield-Gabrieli, 2021; http://www.nitrc.org/projects/conn), including regression of principal components derived from white matter (five components) and cerebrospinal fluid (five components), 12 motion-related regressors (six realignment parameters and their temporal derivatives), a constant and linear trend (effect of rest), and a lesion confound regressor. Subject-specific lesion masks were included during denoising to exclude signal originating from lesioned tissue and reduce artifactual connectivity estimates from damaged regions. Temporal band-pass filtering (0.008–0.09 Hz) was applied simultaneously with nuisance regression.

### 2.5 Language network regions of interest

**Fig. 3:**
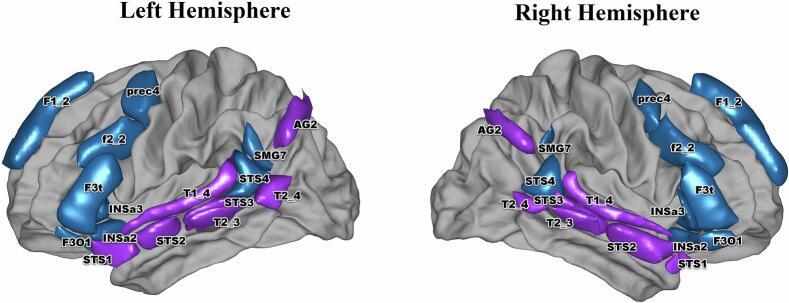
**Locations of the ROIs in the left and right hemispheres**. ROIs belonging to the dorsal stream are shown in blue, and ROIs belonging to the ventral stream are shown in purple. Areas defined by AICHA atlas: prec4 – Precentral sulcus-4; F1_2 – Superior frontal gyrus-2; f2_2 – Inferior frontal sulcus-2; F3t – Inferior frontal pars triangularis gyrus-1; F3O1 – Inferior frontal pars opercularis gyrus-1; INSa2 – Anterior insula gyrus-2 (ventral part); INSa3 – Anterior insula gyrus-3 (dorsal part); STS4 – Superior temporal sulcus-4; SMG7 – Supramarginal gyrus-7; T1_4 – Superior temporal gyrus-4; T2_3 – Middle temporal gyrus-3 (middle part); T2_4 – Middle temporal gyrus-4 (posterior part); STS1 – Superior temporal sulcus-1 (anterior part); STS2 – Superior temporal sulcus-2 (middle part); STS3 – Superior temporal sulcus-3 (posterior part); AG2 – Angular gyrus-2.

Regions of interest (ROIs) were defined based on dorsal and ventral stream language network nodes using the AICHA atlas (Joliot et al., 2015), following the approach of Labache et al. (2019). Although the ventral stream is bilateral, right hemisphere homologs of dorsal stream regions were also extracted to permit cross-hemispheric analyses. ROIs are illustrated in Fig. 3 and listed in Supplementary Table A.2.

### 2.6 Functional connectivity analyses

Functional connectivity analyses were conducted using the CONN Toolbox (version 18b; Nieto-Castanon and Whitfield-Gabrieli, 2021; http://www.nitrc.org/projects/conn). At the first level, ROI-to-ROI functional connectivity matrices (32 × 32) were computed for each participant using Fisher z-transformed bivariate correlation coefficients.

#### 2.6.1 Network-level functional connectivity analyses

Network-level functional connectivity values were calculated for each participant as the mean of all unique pairwise ROI-to-ROI connections within the dorsal stream, ventral stream, and the whole language network (dorsal + ventral streams). Separate general linear models (GLMs) were conducted for the dorsal stream, ventral stream, and whole language network in R using the base stats package. Predictors included aphasia type, severity (WAB-AQ), and the type × severity interaction. Additional models using conduction aphasia as the reference group were conducted to evaluate pairwise subtype differences not directly estimated in the original model parameterization. Covariates included lesion volume, age, sex, days between WAB assessment and rs-fMRI acquisition, time post-stroke at scanning, and maximum absolute head motion. When interaction effects were non-significant, reduced main-effects models excluding the interaction term were evaluated.

#### 2.6.2 ROI-to-ROI functional connectivity analyses

Because network-level analyses summarize functional connectivity across multiple ROIs within a network or stream, they may obscure heterogeneity across individual connections. We therefore also conducted ROI-to-ROI analyses using second-level GLMs in CONN to characterize connection-level differences between groups and functional connectivity patterns within groups. For all models, statistical significance was determined using a two-tailed threshold of *p* < .05 with seed-level FDR correction.

##### 2.6.2.1 Between-group ROI-to-ROI Analyses

Between-group analyses were conducted to identify specific ROI-to-ROI connections that differed across aphasia type and severity groups while adjusting for age, sex, WAB score, days between WAB assessment and rs-fMRI acquisition, lesion volume, time post-stroke at scanning, and maximum absolute head motion.

##### 2.6.2.2 Within-group ROI-to-ROI analyses

Within-group analyses were conducted separately for each group to characterize patterns of functional connectivity organization within aphasia type and severity groups. Two models were implemented. Model 1 tested whether ROI-to-ROI connectivity differed significantly from zero, identifying connections showing consistent positive or negative coupling within a group. Model 2 tested whether ROI-to-ROI connectivity varied as a function of aphasia severity within each type by regressing connectivity values on WAB-AQ scores. Because lower WAB-AQ scores reflect greater aphasia severity, positive associations indicate stronger connectivity in participants with milder aphasia, whereas negative associations indicate stronger connectivity in participants with more severe aphasia. Both models adjusted for the same covariates described above.

To further contextualize within-group functional connectivity organization at the individual participant level, subject-specific 32 × 32 ROI-to-ROI connectivity matrices were thresholded at |z| > 0.3. Representative connectivity matrices from three participants per group (selected to reflect low, mid, and high WAB-AQ scores) were visualized to illustrate variability in functional organization. Because visualizing all individual matrices was not feasible given the sample size, connectivity density was quantified by extracting, for each participant, the total number of suprathreshold positive and negative connections across all ROI pairs. Paired Wilcoxon signed-rank tests were used to compare the number of suprathreshold positive and negative connections within each group.

#### 2.6.3 Group-level lesion overlap analyses

Lesion overlap analyses were conducted to identify regions with consistent structural damage within each group and provide anatomical context for interpreting ROI-to-ROI functional connectivity patterns. MRIcron (Rorden and Brett, 2000) was used to compute descriptive statistics of lesion overlap in each ROI within each group. ROIs were considered to show meaningful group-level lesion overlap if (a) at least 50% of participants within a group had lesions involving that ROI and (b) the overlapping lesioned portion of the ROI comprised at least 50 voxels (1 × 1 × 1 mm; ≥ 50 mm^3^). These criteria were applied to avoid interpreting regions with minimal overlap as reflecting reliable group-level lesion patterns.

## 3 Results

### 3.1 Network-level functional connectivity

The type × severity interaction terms were non-significant for the whole language network and ventral stream models. Therefore, reduced main-effects models excluding the interaction term were subsequently evaluated. No significant effects of aphasia type or severity were observed for whole network functional connectivity (dorsal + ventral streams) or ventral stream connectivity (all *ps* > 0.10; Supplementary Tables A.3–A.6).

For the dorsal stream, the interaction model was not significant (*F*(11,56) = 1.79, *p* = .079, adjusted *R*^*2*^ = 0.114) (Supplementary Materials Table A.7), however, the reduced main-effects model was significant (*F*(9,58) = 2.20, *p* = .035, adjusted *R*^*2*^ = 0.139). After controlling for all covariates, patients with conduction aphasia exhibited greater dorsal stream connectivity relative to the anomic group (*β* = 0.096, *p* = .035), whereas no significant differences were observed between the Broca's and anomic groups (*β* = 0.025, *p* = .598) or between the Broca's and conduction groups (*β* = −0.070, *p* = .116). In contrast, aphasia severity did not predict dorsal stream connectivity (*p* = .518) (Supplementary Materials Table A.8).

### 3.2 ROI-to-ROI functional connectivity

#### 3.2.1 Between group ROI-to-ROI analyses

**Fig. 4:**
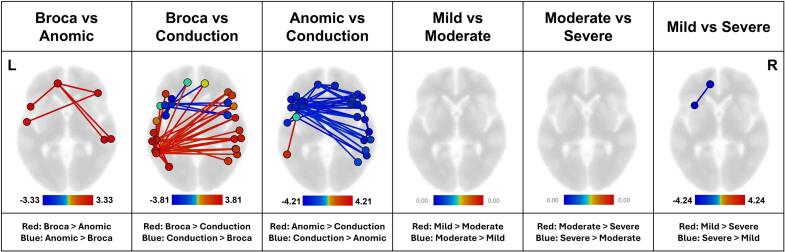
**Between-group ROI-to-ROI functional connectivity comparisons.** Significant between-group differences in ROI-to-ROI functional connectivity are shown for aphasia type and severity comparisons. Warmer colors (red) indicate stronger functional connectivity effects in the first group listed for each comparison, whereas cooler colors (blue) indicate stronger connectivity effects in the second group. The color bar reflects the minimum and maximum T-statistics derived from the second-level ROI-to-ROI analyses.

For aphasia type, the Broca's group demonstrated stronger functional connectivity in one left hemisphere and four interhemispheric connections relative to the anomic group (Fig. 4; Supplementary Materials Table A.9). Compared to the conduction aphasia group, the Broca's group showed stronger connectivity in 16 left hemisphere and 23 interhemispheric connections, whereas the conduction group demonstrated stronger connectivity than the Broca's group in six left hemisphere and five interhemispheric connections (Fig. 4; Supplementary Materials Table A.10). Finally, relative to the anomic group, the conduction group demonstrated stronger functional connectivity in three right hemisphere, nine left hemisphere, and 36 interhemispheric connections, whereas the anomic group showed only one stronger left hemisphere connection relative to the conduction group (Fig. 4; Supplementary Materials Table A.11).

Second, we compared functional connectivity across aphasia severity groups. No significant differences in functional connectivity were observed between the mild and moderate groups or between the moderate and severe groups (Fig. 4). However, comparison of the mild and severe groups revealed one stronger left-hemisphere functional connection in the severe group (Fig. 4; Supplementary Materials Table A.12).

#### 3.2.2 Within-group ROI-to-ROI analyses: Aphasia type

##### 3.2.2.1 Aphasia collapsed across type

**Fig. 5:**
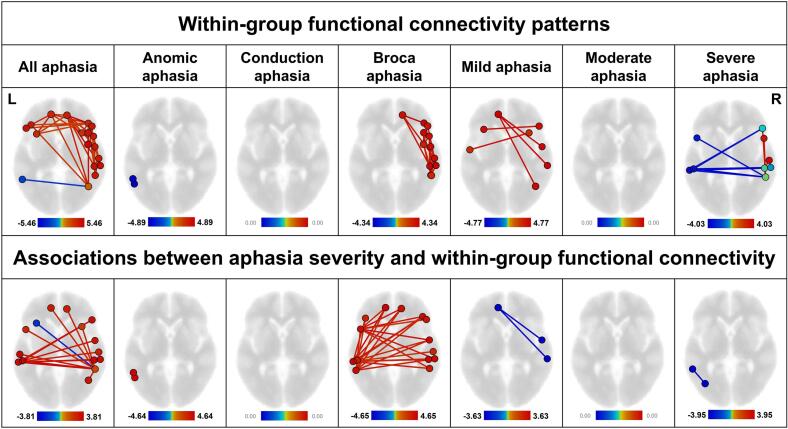
**Within-group ROI-to-ROI functional connectivity organization.** The top panel shows group-level ROI-to-ROI functional connectivity patterns for each aphasia type and severity (Model 1), and the bottom panel shows associations between aphasia severity and ROI-to-ROI functional connectivity within each aphasia type (Model 2). The color bar reflects the range of T-statistics obtained from the second-level ROI-to-ROI analyses. Warmer colors (red) indicate stronger positive effects, cooler colors (blue) indicate stronger negative effects, and intermediate colors indicate effects closer to zero.

Across all PWA, Model 1 revealed significant mean functional connectivity within the left hemisphere (three positive connections), within the right hemisphere (30 positive connections), and between hemispheres (nine positive and one negative connection) (Fig. 5 and Supplementary Materials Table A.13). In Model 2, higher WAB-AQ scores (reflecting milder aphasia) were associated with stronger functional connectivity in three right hemisphere and 11 interhemispheric connections, whereas one interhemispheric connection demonstrated the opposite pattern, such that greater aphasia severity was associated with stronger connectivity (Fig. 5 and Supplementary Materials Table A.14).

##### 3.2.2.2 Anomic Aphasia

**Fig. 6:**
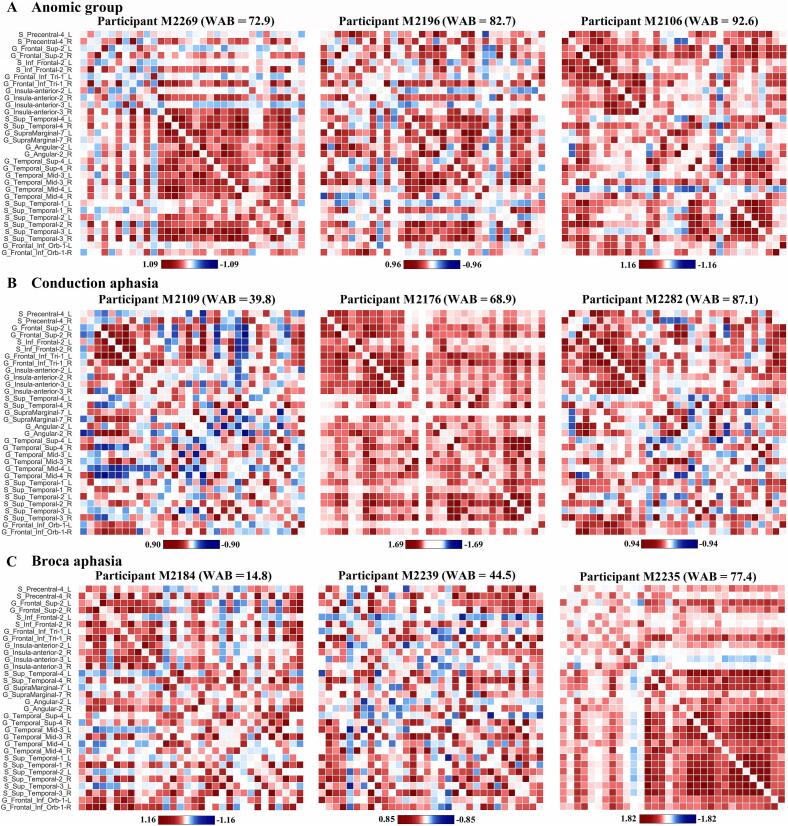
**Representative individual functional connectivity patterns from each aphasia type**. For each group, three participants are shown, ordered by aphasia severity: most severe (left), mid-range (middle), and least severe (right). The color bar limits indicate the minimum and maximum *Z*-values (Fisher-z correlations) within each individual subject. Dark red represents the strongest positive correlation, dark blue the strongest negative correlation, and white values correspond to correlations near zero.

Model 1 identified one significant negative functional connection between left temporal and parietal regions (Fig. 5; Supplementary Materials Table A.15). In Model 2, this same connection was positively associated with aphasia severity, such that individuals with milder aphasia demonstrated stronger connectivity between these regions (Fig. 5; Supplementary Materials Table A.16). Individual variability analyses revealed predominantly positive connectivity patterns across participants (positive connections: *M* = 178.53, *SD* = 60.73; negative connections: *M* = 4.95, *SD* = 3.19; Wilcoxon signed-rank test: *Z* = −3.82, *p* < .001; Fig. 6A). Group-level lesion overlap analysis revealed minimal common lesion involvement, consistent with substantial variability in lesion volume within the group (Table 1). At the 50% threshold, some overlap was observed in the left superior temporal and supramarginal regions; however, at the 100% threshold, no single cortical area was consistently affected across participants (Fig. 1B).

##### 3.2.2.3 Conduction aphasia

Model 1 did not identify any ROI-to-ROI connections that significantly differed from zero, and Model 2 revealed no significant associations between aphasia severity and ROI-to-ROI connectivity. Individual variability analyses revealed predominantly positive connectivity patterns across participants (positive connections: *M* = 202.22, *SD* = 124.28; negative connections: *M* = 9.11, *SD* = 6.35; Wilcoxon signed-rank test: *Z* = −2.666, *p* = .008; Fig. 6B). Despite variability in lesion volume across participants (Table 1), group-level lesion overlap analyses indicated consistent structural involvement of left inferior parietal and left superior and middle temporal regions at the 50% threshold (Fig. 1C). At the 100% threshold, lesion distribution was broader and additionally included left frontal and occipital regions (Fig. 1C).

##### 3.2.2.4 Broca's aphasia

Model 1 identified 26 positive ROI-to-ROI connections within the right hemisphere but no significant left hemisphere connections (Fig. 5; Supplementary Materials Table A.17). In Model 2, higher WAB-AQ scores (reflecting milder aphasia) were associated with stronger functional connectivity across six left hemisphere and 18 interhemispheric connections, indicating enhanced left hemisphere connectivity and interhemispheric coupling in individuals with milder aphasia (Fig. 5; Supplementary Materials Table A.18). Individual variability analyses demonstrated predominantly positive connectivity patterns across participants (positive connections: *M* = 158.35, *SD* = 72.61; negative connections: *M* = 8.08, *SD* = 7.86; Wilcoxon signed-rank test: *Z* = −5.511, *p* < .001; Fig. 6C). Group-level lesion overlap analyses indicated consistent structural involvement of the left inferior and middle frontal regions, rolandic operculum, precentral and postcentral sulci, insula, superior temporal gyrus, and supramarginal gyrus at the 50% overlap threshold (Fig. 1D). At the 100% overlap threshold, lesion distribution additionally involved broader left temporal and parietal regions, consistent with the generally large lesion volumes observed within this group (Table 1).

#### 3.2.3 Within-group ROI-to-ROI analyses: aphasia severity

Although Model 2 examined severity effects within type analyses, we additionally stratified participants by aphasia severity to determine whether functional connectivity patterns differed systematically across severity groups independent of aphasia classification.

##### 3.2.3.1 No aphasia

**Fig. 7:**
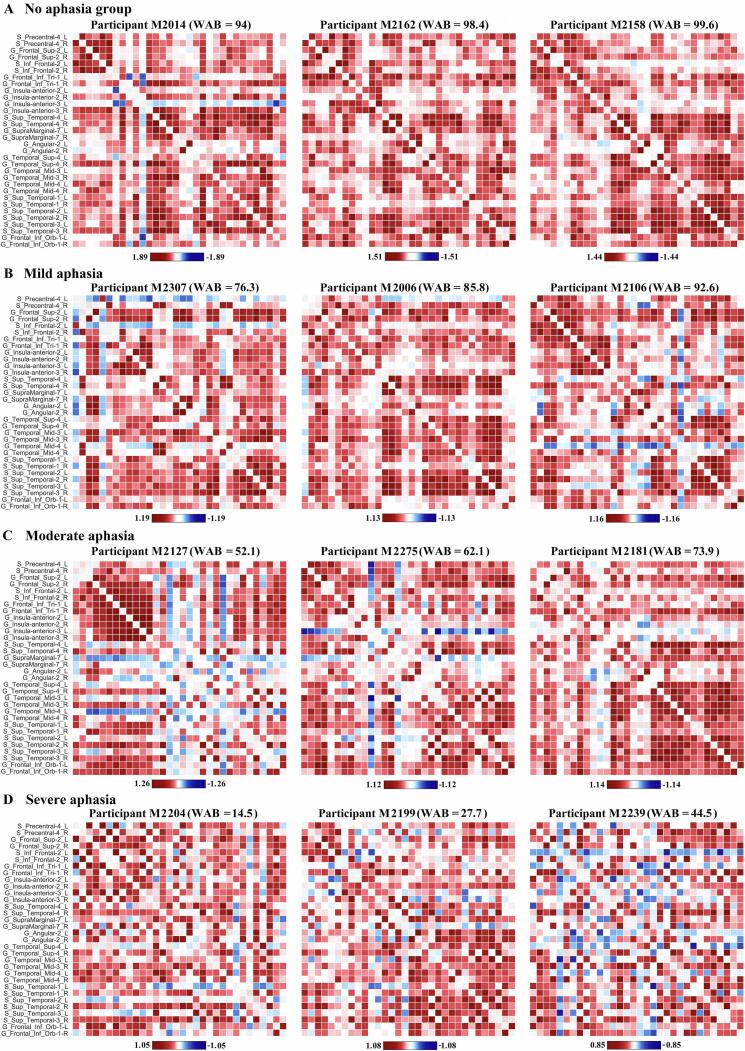
**Representative individual functional connectivity patterns from each severity group**. For each group, three participants are shown, ordered by aphasia severity: most severe (left), mid-range (middle), and least severe (right). The color bar limits indicate the minimum and maximum Z-values (Fisher-z correlations) within each individual subject. Dark red represents the strongest positive correlation, dark blue the strongest negative correlation, and white values correspond to correlations near zero.

Model 1 did not identify any ROI-to-ROI connections that significantly differed from zero, and Model 2 revealed no significant associations between aphasia severity and ROI-to-ROI connectivity. Individual variability analyses showed significantly more positive than negative connections across participants (positive connections: *M* = 219.92, *SD* = 72.25; negative connections: *M* = 8.83, *SD* = 10.40; Wilcoxon signed-rank test: *Z* = −3.059, *p* = .002; Fig. 7A). Group-level lesion overlap analysis revealed common involvement primarily limited to left hemisphere white-matter tracts, with no single cortical area consistently affected across participants (Fig. 2B). Consistent with these findings, lesion volume also demonstrated substantial variability within the group (Table 1).

##### 3.2.3.2 Mild Aphasia

Model 1 revealed five positive interhemispheric ROI-to-ROI connections. In Model 2, two interhemispheric connections were negatively associated with aphasia severity, indicating stronger connectivity in individuals with more severe aphasia (Fig. 5; Supplementary Materials Table A.19 and Table A.20). Individual variability analyses demonstrated predominantly positive connectivity patterns across participants (positive connections: *M* = 180.88, *SD* = 67.66; negative connections: *M* = 5.84, *SD* = 3.53; Wilcoxon signed-rank test: *Z* = −4.286, *p* < .001; Fig. 7B). Group-level lesion overlap analysis revealed minimal common lesion involvement. At the 50% overlap threshold, limited overlap was observed in the left superior temporal and supramarginal regions. At the 100% overlap threshold, lesions were distributed across a wide range of left cortical regions, with no single area consistently affected across participants (Fig. 2C). Consistent with this pattern, lesion volume within this group demonstrated substantial variability (Table 1).

##### 3.2.3.3 Moderate aphasia

Model 1 did not identify any ROI-to-ROI connections that significantly differed from zero, and Model 2 revealed no significant associations between aphasia severity and ROI-to-ROI connectivity. Individual variability analyses demonstrated significantly more positive than negative connections across participants (positive connections: *M* = 184.44, *SD* = 85.07; negative connections: *M* = 8.20, *SD* = 7.11; Wilcoxon signed-rank test: *Z* = −4.286, *p* < .001; Fig. 7C). Group-level lesion overlap analysis indicated common involvement of the left frontal, temporal, and parietal language regions at the 50% overlap threshold. At the 100% overlap threshold, lesion distribution was broader within these cortical regions and additionally involved occipital regions (Fig. 2D). Although the Moderate aphasia group demonstrated relatively consistent lesion topography, lesion volumes remained highly variable across participants (Table 1).

##### 3.2.3.4 **Severe** aphasia

Model 1 identified five positive ROI-to-ROI connections within the right hemisphere and nine negative interhemispheric connections but no significant left hemisphere connections (Fig. 5; Supplementary Materials Table A.21). In Model 2, connectivity between the left angular gyrus and left superior temporal gyrus was negatively associated with WAB-AQ scores, indicating stronger functional coupling between these regions in individuals with more severe aphasia (Fig. 5; Supplementary Materials Table A.22). Individual variability analyses demonstrated predominantly positive connectivity patterns across participants (positive connections: *M* = 139.56, *SD* = 69.78; negative connections: *M* = 9.33, *SD* = 10.19; Wilcoxon signed-rank test: *Z* = −4.541, *p* < .001; Fig. 7D). Group-level lesion overlap analyses revealed involvement of left frontal, temporal, and parietal language regions at the 50% overlap threshold, with lesion distribution becoming broader and extending into the left occipital lobe at the 100% overlap threshold (Fig. 2E). Consistent with these overlap patterns, the severe aphasia group had relatively large lesion volumes across participants (Table 1).

## 4 Discussion

Patterns of language network functional connectivity in post-stroke aphasia vary widely across studies, and aphasia type may provide additional context for understanding this heterogeneity. In the present study, we first examined network-level functional connectivity across aphasia type and severity groups within the dorsal stream, ventral stream, and whole language network, including the interaction between aphasia type and severity. We then conducted ROI-to-ROI analyses to characterize connection-level (edgewise) connectivity patterns within aphasia types and across severity groups. Compared with severity-based groupings, type-based analyses generally revealed more differentiated and spatially interpretable connectivity patterns, likely reflecting greater convergence in language profiles and lesion topography within aphasia types. These findings suggest that aphasia type may provide additional context for understanding variability in post-stroke language network connectivity that may not be fully captured by severity measures alone.

At the network level, regression models testing aphasia type, severity, and their interaction demonstrated relatively limited explanatory power for global connectivity measures, particularly within the ventral stream and whole language network. One possible explanation is that averaging connectivity across all ROI-to-ROI pairs may obscure heterogeneous or connection-specific effects by combining positive, negative, and near-zero connectivity patterns into a single summary metric that may be attenuated. Nevertheless, dorsal stream connectivity demonstrated greater sensitivity to aphasia type than to overall severity, suggesting that aphasia type may provide additional context for understanding heterogeneity in residual language network organization beyond severity measures alone. However, this finding should be interpreted cautiously, as only a single subtype comparison reached significance (conduction > anomic). Given the substantial lesion heterogeneity observed within the anomic group, the higher dorsal stream connectivity observed in conduction aphasia may reflect greater convergence in lesion distributions and language profiles within the conduction group rather than a connectivity pattern specific to conduction aphasia itself. Future studies are needed to determine whether this pattern replicates in larger samples and reflects a reliable subtype-related difference in dorsal stream connectivity.

Although network-level analyses revealed relatively limited effects within each stream and the whole language network, ROI-to-ROI (edgewise) analyses demonstrated that aphasia severity modulated functional connectivity patterns within the Broca's aphasia group. Specifically, milder aphasia was associated with stronger left hemisphere and interhemispheric connectivity than more severe aphasia. These severity-related connectivity patterns are consistent with the possibility that variability in residual network integrity is associated with differences in language performance even among individuals with the same aphasia type. Consistent with prior work demonstrating the importance of spared language networks and network hub integrity for aphasia outcomes (Gleichgerrcht et al., 2015; Saur et al., 2006), these findings suggest that better language performance is associated with stronger preservation of left hemisphere language connectivity together with more efficient interhemispheric communication.

Additional between-group ROI-to-ROI analyses revealed systematic connectivity differences across all aphasia type comparisons. Previous work using lesion-controlled resting-state network analyses reported reduced functional connectivity within a bilateral dorsal stream subnetwork in Broca's aphasia relative to anomic aphasia, including sensorimotor, temporal, and insular regions (Riccardi et al., 2024). In contrast, we observed relatively few connectivity differences between these groups, with the significant effects primarily involving stronger interhemispheric connections between left frontal and right temporal or right inferior frontal regions, as well as one left intrahemispheric frontal–temporal connection in the Broca's group. One possible explanation for this discrepancy is differences in sample composition, lesion characteristics, network definitions, and analytical approach across studies. Inclusion of individuals with conduction aphasia allowed us to extend our comparisons beyond those examined by Riccardi et al. Compared with the conduction group, the Broca's group demonstrated stronger connectivity within left hemisphere dorsal and ventral stream regions, as well as stronger interhemispheric connectivity between left hemisphere language regions and right hemisphere dorsal and ventral stream regions. In contrast, the conduction group demonstrated stronger connectivity primarily within left dorsal stream regions, particularly involving the anterior insula, as well as several cross-hemispheric frontal and temporal connections. Similarly, conduction aphasia demonstrated substantially more widespread left hemisphere, right hemisphere, and interhemispheric connectivity than anomic aphasia, whereas the anomic group demonstrated only a single stronger left hemisphere connection.

Severity-based comparisons identified only a single significant connection between the mild and severe groups. Within-group ROI-to-ROI analyses similarly revealed limited connectivity effects in several groups characterized by considerable lesion and clinical heterogeneity, particularly the no aphasia, mild, and moderate groups. In contrast, the severe aphasia group, which was characterized by more convergent lesion distributions and was approximately 80% Broca's aphasia, exhibited more consistent connectivity patterns across individuals. These findings suggest that weak group-level effects within severity classifications likely reflect heterogeneity in lesion location, language profile, and residual network organization rather than an absence of meaningful connectivity patterns. Consistent with this interpretation, subject-level connectivity patterns were predominantly positive across language regions, even within the more clinically heterogeneous severity groups. This interpretation is further supported by previous work demonstrating substantial inter-individual variability in functional connectivity organization even among healthy adults (Baldassarre et al., 2012; Mueller et al., 2013).

Taken together, the present findings suggest that variability in residual functional connectivity in post-stroke aphasia is shaped by a combination of lesion characteristics, language profile, impairment severity, and residual network integrity. Groups characterized by greater convergence in lesion distribution and clinical presentation exhibited more consistent connectivity patterns, whereas groups with greater anatomical and behavioral heterogeneity showed weaker group-level effects despite evidence of preserved connectivity at the individual level. Accordingly, the observed subtype-related connectivity patterns should not be interpreted as evidence that aphasia type represents a biologically distinct entity independent of lesion anatomy. Rather, aphasia type may provide useful clinical context for understanding variability in post-stroke language network organization because it captures partially shared lesion distributions and language profiles that are not fully reflected by severity measures alone. In this context, aphasia type may aid interpretation of post-stroke heterogeneity in both neuroimaging and behavioral studies, particularly when detailed lesion characterization or comprehensive behavioral language profiles are unavailable.

## 5 Limitations and future directions

While informative, these results should be interpreted in light of several limitations. First, data for this study were drawn from an open-source repository, which constrained participant characterization to variables available in the dataset. Although basic demographic information (e.g., age, sex) was available, language characterization was limited to aphasia type and severity, with little information on specific language abilities. As a result, we were unable to examine how functional connectivity patterns related to discrete language behaviors. Future work should incorporate detailed behavioral measures, as different language domains (e.g., semantic, syntactic, phonological processing) are likely supported by partially distinct neural networks.

A second limitation concerns the lack of information regarding participants' rehabilitation histories, and it is possible that some participants received therapy despite being in the chronic stage of recovery. Rehabilitation has been shown to modulate functional connectivity patterns following stroke (Arun et al., 2020; Fan et al., 2015; Park et al., 2011; Xu et al., 2014). The potential influence of rehabilitation may be particularly relevant for participants with longer intervals between rs-fMRI acquisition and WAB assessment.

An additional limitation concerns the network-level analyses, which summarized functional connectivity by averaging ROI-to-ROI connections within the dorsal stream, ventral stream, and whole language network. Although this approach reduced the dimensionality of the data and enabled parsimonious testing of broad network-level hypotheses, averaging connectivity across multiple connections may have obscured regionally specific effects. Consistent with this possibility, several findings emerged only in the ROI-to-ROI analyses. Future studies may benefit from larger samples that permit more comprehensive modeling of connection-level functional connectivity while adequately controlling for multiple comparisons.

Although the overall sample size was moderately large (*n* = 89), stratifying participants by aphasia type and severity resulted in smaller subgroup sample sizes, which may have reduced statistical power and constrained the strength of subgroup-level inferences. Future studies should leverage larger, well-characterized samples with more detailed behavioral and rehabilitation data. More balanced aphasia type groups may also permit analyses within more homogeneous lesion profiles (Wilson et al., 2023), facilitating clearer interpretation of structure–function relationships and enabling integration of lesion-derived disconnection measures with resting-state functional connectivity analyses across aphasia subgroups. In addition, longitudinal designs spanning the acute through chronic stages of recovery may further elucidate how changes in functional connectivity over time relate to language recovery in aphasia.

## 6 Conclusions

In summary, the present findings demonstrate that patterns of functional connectivity following left-hemisphere stroke are not uniform across aphasia, but instead reflect interactions between clinical profile, lesion distribution, and aphasia severity. Resting-state functional connectivity varied across both aphasia type and severity groups, with type-based analyses generally revealing more differentiated connectivity findings than severity-based groupings alone. Severity primarily modulated connectivity within individual aphasia types rather than independently accounting for variability in network connectivity across participants. Together, these findings suggest that aphasia type may provide useful complementary clinical context for understanding heterogeneity in post-stroke language network connectivity alongside severity measures. More broadly, this work highlights the importance of accounting for clinical and lesion heterogeneity in network-level studies of aphasia and motivates future research integrating aphasia type, lesion characteristics, and individual network profiles to better characterize the neural basis of post-stroke language impairment.

## CRediT authorship contribution statement

**Svetlana V. Kuptsova:** Writing – review & editing, Writing – original draft, Visualization, Methodology, Formal analysis, Data curation, Conceptualization. **Arianna N. LaCroix:** Writing – review & editing, Supervision, Project administration.

## Funding

This research was supported by Purdue University.

## Declaration of competing interest

The authors declare that they have no known competing financial interests or personal relationships that could have appeared to influence the work reported in this paper.

## Data Availability

The authors do not have permission to share data.
