# Supplementary material for: Language network functional connectivity varies by aphasia type and severity
Source: Neuroimage Clin. 2026 Jul 3;51:104030. doi: 10.1016/j.nicl.2026.104030 (PMC13356682; doi:10.1016/j.nicl.2026.104030)
Supplement: Supplementary file 1 — Supplementary material [file mmc1.docx]

**Table A.1. Individual characteristics of participants**

| **#** | **participant_id** | **sex** | **age_at_stroke** | **rs_days** | **wab_days** | **days_between** | **wab_aq** | **wab_type** | **voxel size_norm** | **voxel_size_native** | **severity** |
| --- | --- | --- | --- | --- | --- | --- | --- | --- | --- | --- | --- |
| 1 | sub-M2005 | F | 31 | 4334 | 4334 | 0 | 54.8 | Broca | 99766 | 77168 | Moderate |
| 2 | sub-M2006 | M | 46 | 5432 | 5415 | 17 | 85.8 | Anomic | 121063 | 77691 | Mild |
| 3 | sub-M2014 | M | 56 | 1959 | 1958 | 1 | 94 | None | 96260 | 68863 |  |
| 4 | sub-M2029 | M | 43 | 2706 | 2709 | 3 | 40.6 | Broca | 271769 | 218835 | Severe |
| 5 | sub-M2036 | M | 50 | 2722 | 2722 | 0 | 72.2 | Broca | 196410 | 166534 | Moderate |
| 6 | sub-M2040 | M | 49 | 2439 | 2416 | 23 | 52.9 | Broca | 277065 | 201976 | Moderate |
| 7 | sub-M2049 | F | 67 | 2530 | 2535 | 5 | 63.4 | Conduction | 19049 | 14136 | Moderate |
| 8 | sub-M2059 | M | 57 | 3349 | 3349 | 0 | 53.7 | Broca | 162799 | 119556 | Moderate |
| 9 | sub-M2072 | M | 41 | 3004 | 3004 | 0 | 30.5 | Broca | 210781 | 176812 | Severe |
| 10 | sub-M2075 | F | 40 | 529 | 529 | 0 | 94.2 | None | 33102 | 25720 |  |
| 11 | sub-M2099 | M | 48 | 1952 | 1952 | 0 | 56.3 | Broca | 409927 | 264827 | Moderate |
| 12 | sub-M2102 | M | 47 | 1400 | 1400 | 0 | 86 | Conduction | 94928 | 75552 | Mild |
| 13 | sub-M2106 | M | 58 | 2332 | 2422 | 90 | 92.6 | Anomic | 75547 | 57481 | Mild |
| 14 | sub-M2109 | F | 66 | 1170 | 1160 | 10 | 39.8 | Conduction | 106700 | 81930 | Severe |
| 15 | sub-M2127 | M | 58 | 435 | 435 | 0 | 52.1 | Broca | 272460 | 214905 | Moderate |
| 16 | sub-M2141 | F | 72 | 2539 | 2538 | 1 | 99.1 | None | 239 | 273 |  |
| 17 | sub-M2143 | F | 61 | 2871 | 2840 | 31 | 99.2 | None | 3249 | 2825 |  |
| 18 | sub-M2146 | F | 29 | 4696 | 4696 | 0 | 96.8 | None | 82004 | 65287 |  |
| 19 | sub-M2149 | F | 62 | 677 | 676 | 1 | 98.6 | None | 200 | 251 |  |
| 20 | sub-M2155 | F | 60 | 814 | 813 | 1 | 98.9 | None | 1564 | 1404 |  |
| 21 | sub-M2156 | F | 31 | 2653 | 2652 | 1 | 96.7 | None | 78718 | 44910 |  |
| 22 | sub-M2158 | F | 50 | 404 | 403 | 1 | 99.6 | None | 1326 | 1408 |  |
| 23 | sub-M2159 | M | 71 | 380 | 349 | 31 | 99.1 | None | 30789 | 23176 |  |
| 24 | sub-M2160 | F | 40 | 4825 | 4822 | 3 | 96.4 | None | 128141 | 64588 |  |
| 25 | sub-M2162 | M | 59 | 240 | 240 | 0 | 98.4 | None | 2844 | 2415 |  |
| 26 | sub-M2165 | M | 51 | 1470 | 1456 | 14 | 20.1 | Broca | 367707 | 279986 | Severe |
| 27 | sub-M2168 | M | 55 | 1445 | 1445 | 0 | 71.7 | Broca | 137828 | 116666 | Moderate |
| 28 | sub-M2170 | M | 42 | 660 | 660 | 0 | 91.8 | Anomic | 25650 | 23892 | Mild |
| 29 | sub-M2172 | M | 68 | 476 | 476 | 0 | 77.2 | Anomic | 75623 | 63165 | Mild |
| 30 | sub-M2173 | M | 71 | 884 | 893 | 9 | 57.8 | Broca | 104218 | 90698 | Moderate |
| 31 | sub-M2175 | M | 52 | 703 | 816 | 113 | 23.7 | Global | 93788 | 77103 | Severe |
| 32 | sub-M2176 | M | 66 | 927 | 927 | 0 | 68.9 | Conduction | 148175 | 122670 | Moderate |
| 33 | sub-M2177 | M | 70 | 371 | 344 | 27 | 91.3 | Anomic | 28364 | 22272 | Mild |
| 34 | sub-M2178 | M | 75 | 408 | 408 | 0 | 30.6 | Broca | 115915 | 68234 | Severe |
| 35 | sub-M2179 | M | 46 | 2515 | 2515 | 0 | 25.2 | Global | 128990 | 105869 | Severe |
| 36 | sub-M2181 | M | 42 | 502 | 502 | 0 | 73.9 | Broca | 80351 | 57955 | Moderate |
| 37 | sub-M2182 | F | 27 | 802 | 802 | 0 | 79.2 | Conduction | 251492 | 184988 | Mild |
| 38 | sub-M2183 | M | 75 | 442 | 442 | 0 | 80.3 | Anomic | 47628 | 39169 | Mild |
| 39 | sub-M2184 | M | 45 | 358 | 358 | 0 | 14.8 | Broca | 98239 | 81093 | Severe |
| 40 | sub-M2186 | F | 36 | 446 | 446 | 0 | 65.5 | Broca | 202093 | 125959 | Moderate |
| 41 | sub-M2196 | F | 68 | 386 | 386 | 0 | 82.7 | Anomic | 93263 | 64985 | Mild |
| 42 | sub-M2197 | M | 68 | 1473 | 1473 | 0 | 22.8 | Broca | 190373 | 132591 | Severe |
| 43 | sub-M2198 | M | 46 | 1073 | 1073 | 0 | 31.3 | Global | 242178 | 195590 | Severe |
| 44 | sub-M2199 | F | 66 | 671 | 671 | 0 | 27.7 | Broca | 187494 | 113518 | Severe |
| 45 | sub-M2200 | M | 58 | 783 | 783 | 0 | 31.4 | Broca | 132699 | 94675 | Severe |
| 46 | sub-M2202 | M | 55 | 423 | 423 | 0 | 27.4 | Broca | 147018 | 98396 | Severe |
| 47 | sub-M2204 | F | 63 | 577 | 577 | 0 | 14.5 | Global | 245447 | 162028 | Severe |
| 48 | sub-M2207 | F | 63 | 3014 | 3014 | 0 | 91.3 | Anomic | 30125 | 23089 | Mild |
| 49 | sub-M2210 | M | 64 | 4388 | 4388 | 0 | 89.4 | Anomic | 15943 | 13642 | Mild |
| 50 | sub-M2211 | M | 60 | 453 | 453 | 0 | 57.9 | Broca | 187704 | 156179 | Moderate |
| 51 | sub-M2212 | F | 46 | 6327 | 6327 | 0 | 64 | Broca | 161545 | 113538 | Moderate |
| 52 | sub-M2213 | F | 71 | 1743 | 1743 | 0 | 25.4 | Broca | 241832 | 156968 | Severe |
| 53 | sub-M2214 | M | 74 | 362 | 362 | 0 | 85 | Anomic | 65561 | 58916 | Mild |
| 54 | sub-M2216 | F | 56 | 2015 | 2015 | 0 | 67 | Broca | 165830 | 111527 | Moderate |
| 55 | sub-M2220 | F | 58 | 359 | 359 | 0 | 57.7 | Conduction | 91711 | 62346 | Moderate |
| 56 | sub-M2223 | F | 37 | 644 | 644 | 0 | 92.2 | Anomic | 138012 | 88442 | Mild |
| 57 | sub-M2226 | M | 56 | 311 | 366 | 55 | 69.2 | Wernicke | 176278 | 126394 | Moderate |
| 58 | sub-M2227 | M | 58 | 424 | 424 | 0 | 78.2 | TranscorticalMotor | 47725 | 40232 | Mild |
| 59 | sub-M2228 | F | 65 | 456 | 518 | 62 | 19.9 | Global | 317611 | 196529 | Severe |
| 60 | sub-M2232 | F | 64 | 492 | 492 | 0 | 90.4 | Anomic | 82256 | 60646 | Mild |
| 61 | sub-M2235 | M | 61 | 398 | 398 | 0 | 77.4 | Broca | 130350 | 108583 | Mild |
| 62 | sub-M2239 | M | 40 | 1609 | 1609 | 0 | 44.5 | Broca | 352079 | 296350 | Severe |
| 63 | sub-M2245 | M | 58 | 6526 | 6580 | 54 | 32.4 | Broca | 155238 | 144698 | Severe |
| 64 | sub-M2260 | M | 46 | 817 | 810 | 7 | 40.1 | Broca | 411011 | 267259 | Severe |
| 65 | sub-M2265 | F | 76 | 849 | 849 | 0 | 76.9 | TranscorticalMotor | 109002 | 70823 | Mild |
| 66 | sub-M2266 | F | 62 | 1380 | 1380 | 0 | 62.9 | Conduction | 119354 | 81401 | Moderate |
| 67 | sub-M2268 | M | 67 | 344 | 344 | 0 | 31.5 | Broca | 313391 | 250101 | Severe |
| 68 | sub-M2269 | F | 61 | 767 | 731 | 36 | 72.9 | Anomic | 304821 | 219644 | Moderate |
| 69 | sub-M2271 | M | 72 | 207 | 207 | 0 | 19.4 | Broca | 204925 | 166789 | Severe |
| 70 | sub-M2275 | M | 47 | 948 | 924 | 24 | 62.1 | Broca | 232968 | 217272 | Moderate |
| 71 | sub-M2276 | M | 67 | 1364 | 1364 | 0 | 19.6 | Broca | 161419 | 150143 | Severe |
| 72 | sub-M2278 | M | 70 | 388 | 388 | 0 | 44 | Broca | 104257 | 72599 | Severe |
| 73 | sub-M2279 | M | 78 | 272 | 272 | 0 | 81.9 | Anomic | 55275 | 44599 | Mild |
| 74 | sub-M2281 | F | 39 | 430 | 430 | 0 | 92 | Anomic | 73205 | 50267 | Mild |
| 75 | sub-M2282 | M | 63 | 534 | 534 | 0 | 87.1 | Conduction | 69353 | 59124 | Mild |
| 76 | sub-M2285 | F | 62 | 344 | 344 | 0 | 76.4 | Anomic | 40556 | 28167 | Mild |
| 77 | sub-M2287 | M | 53 | 397 | 397 | 0 | 65.3 | Broca | 173413 | 148349 | Moderate |
| 78 | sub-M2290 | M | 75 | 320 | 320 | 0 | 29.7 | Broca | 123284 | 100704 | Severe |
| 79 | sub-M2292 | M | 39 | 1285 | 1247 | 38 | 90.1 | Anomic | 126121 | 101326 | Mild |
| 80 | sub-M2293 | M | 56 | 209 | 209 | 0 | 20.2 | Broca | 279550 | 202506 | Severe |
| 81 | sub-M2295 | M | 58 | 239 | 239 | 0 | 15.5 | Broca | 118473 | 99722 | Severe |
| 82 | sub-M2297 | M | 61 | 6264 | 6264 | 0 | 40.1 | Broca | 199533 | 155617 | Severe |
| 83 | sub-M2299 | M | 64 | 424 | 387 | 37 | 52.5 | Broca | 187883 | 147381 | Moderate |
| 84 | sub-M2300 | M | 41 | 1457 | 1451 | 6 | 80.4 | Anomic | 148220 | 122860 | Mild |
| 85 | sub-M2304 | F | 38 | 262 | 262 | 0 | 55.9 | Broca | 88144 | 62302 | Moderate |
| 86 | sub-M2306 | F | 66 | 707 | 707 | 0 | 59.5 | Broca | 178078 | 130574 | Moderate |
| 87 | sub-M2307 | F | 54 | 371 | 371 | 0 | 76.3 | TranscorticalMotor | 14143 | 11080 | Mild |
| 88 | sub-M2308 | M | 59 | 1705 | 1705 | 0 | 90.4 | Anomic | 53160 | 49242 | Mild |
| 89 | sub-M2309 | M | 64 | 960 | 960 | 0 | 60.6 | Conduction | 77632 | 59517 | Moderate |

Notes: F – female; M – male; rs_days – number of days after stroke of resting state acquisition; wab_type – type of aphasia; wab_days – number of days after stroke of WAB score acquisition; days_between – days between WAB test and resting state; wab_aq – WAB score.

**Table A.2. The list of ROIs**

| AICHA name | Region | Abbreviation | X (mm) | Y (mm) | Z (mm) |
| --- | --- | --- | --- | --- | --- |
| **Dorsal Stream (left hemisphere)** | | | | | |
| S_Precentral-4_L | Precentral sulcus-4 Left | prec4 | -42.2 | 0.7 | 49.9 |
| G_Frontal_Sup-2_L | Superior frontal gyrus-2 Left | F1_2 | -11.9 | 46.5 | 41.4 |
| S_Inf_Frontal-2_L | Inferior frontal sulcus-2 Left | f2_2 | -43.1 | 14.8 | 29.4 |
| G_Frontal_Inf_Tri-1_L | Inferior frontal pars triangularis gyrus-1 Left | F3t | -49.4 | 25.6 | 4.7 |
| G_Frontal_Inf_Orb-1_L | Inferior frontal pars opercularis gyrus-1 Left | F3O1 | -42.2 | 30.5 | -16.9 |
| G_Insula-anterior-2_L | Anterior insula gyrus-2 Left | INSa2 | -33.8 | 16.8 | -12.7 |
| G_Insula-anterior-3_L | Anterior insula gyrus-3 Left | INSa3 | -33.7 | 23.7 | 0.6 |
| S_Sup_Temporal-4_L | Superior temporal sulcus-4 Left | STS4 | -56.5 | -48.4 | 13.4 |
| G_SupraMarginal-7_L | Supramarginal gyrus-7 Left | SMG7 | -55.2 | -51.7 | 25.5 |
| **Homologous ROIs of Dorsal Stream in the Right Hemisphere** | | | | | |
| S_Precentral-4_R | Precentral sulcus-4 Right | prec4_R | 43.7 | 1.0 | 48.4 |
| G_Frontal_Sup-2_R | Superior frontal gyrus-2 Right | F1_2_R | 12.0 | 45.1 | 41.8 |
| S_Inf_Frontal-2_R | Inferior frontal sulcus-2 Right | f2_2_R | 43.9 | 18.5 | 28.4 |
| G_Frontal_Inf_Tri-1_R | Inferior frontal pars triangularis gyrus-1 Right | F3t_R | 49.8 | 28.5 | 5.0 |
| G_Frontal_Inf_Orb-1_R | Inferior frontal pars opercularis gyrus-1 Right | F3O1_R | 44.2 | 33.0 | -14.4 |
| G_Insula-anterior-2_R | Anterior insula gyrus-2 Right | INSa2_R | 34.7 | 18.5 | -12.7 |
| G_Insula-anterior-3_R | Anterior insula gyrus-3 Right | INSa3_R | 36.8 | 24.2 | -0.3 |
| S_Sup_Temporal-4_R | Superior temporal sulcus-4 Right | STS4_R | 54.6 | -45.5 | 14.6 |
| G_SupraMarginal-7_R | Supramarginal gyrus-7 Right | SMG7_R | 55.4 | -45.9 | 33.4 |
| **Ventral Stream (left hemisphere)** | | | | | |
| G_Temporal_Sup-4_L | Superior temporal gyrus-4_ Left | T1_4 | -58.7 | -23.3 | 3.7 |
| G_Temporal_Mid-3_L | Middle temporal gyrus-3_ Left | T2_3 | -61.0 | -35.0 | -4.8 |
| G_Temporal_Mid-4_L | Middle temporal gyrus-4_ Left | T2_4 | -53.1 | -59.4 | 7.0 |
| S_Sup_Temporal-1_L | Superior temporal sulcus-1_ Left | STS1 | -49.7 | 14.0 | -21.5 |
| S_Sup_Temporal-2_L | Superior temporal sulcus-2_ Left | STS2 | -54.9 | -7.2 | -12.8 |
| S_Sup_Temporal-3_L | Superior temporal sulcus-3_ Left | STS3 | -54.7 | -33.0 | -1.7 |
| G_Angular-2_L | Angular gyrus-2_Left | AG2 | -37.5 | -70.4 | 39.5 |
| **Homologous ROIs of Ventral Stream in the Right Hemisphere** | | | | | |
| G_Temporal_Sup-4_R | Superior temporal gyrus-4_ Right | T1_4_R | 60.0 | -20.0 | 2.2 |
| G_Temporal_Mid-3_R | Middle temporal gyrus-3_ Right | T2_3_R | 62.3 | -30.9 | -4.7 |
| G_Temporal_Mid-4_R | Middle temporal gyrus-4_ Right | T2_4_R | 56.8 | -53.2 | 3.0 |
| S_Sup_Temporal-1_R | Superior temporal sulcus-1_ Right | STS1_R | 52.1 | 13.4 | -25.9 |
| S_Sup_Temporal-2_R | Superior temporal sulcus-2_ Right | STS2_R | 54.3 | -2.5 | -15.5 |
| S_Sup_Temporal-3_R | Superior temporal sulcus-3_ Right | STS3_R | 53.1 | -31.9 | -0.3 |
| G_Angular-2_R | Angular gyrus-2_ Right | AG2_R | 44.5 | -62.4 | 36.3 |

## **Table A.3. Combined Language Network (Interaction Model)**

| Predictor | β | SE | t | p |
| --- | --- | --- | --- | --- |
| Intercept | 0.385 | 0.379 | 1.015 | .315 |
| Broca vs Anomic | -0.241 | 0.342 | -0.703 | .485 |
| Conduction vs Anomic | -0.212 | 0.377 | -0.563 | .576 |
| Broca vs Conduction | −0.028 | 0.178 | −0.160 | .874 |
| WAB-AQ | -0.0018 | 0.0040 | -0.449 | .655 |
| Lesion volume | -2.34×10⁻⁷ | 1.76×10⁻⁷ | -1.327 | .190 |
| Age at stroke | 0.00027 | 0.00123 | 0.218 | .829 |
| Sex (Male) | 0.025 | 0.030 | 0.846 | .401 |
| Days between WAB and rs-fMRI | 0.00010 | 0.00091 | 0.112 | .911 |
| Time post-stroke | 1.70×10⁻⁵ | 8.42×10⁻⁶ | 2.019 | .048* |
| Head motion | -0.024 | 0.022 | -1.094 | .279 |
| Broca × WAB-AQ | 0.0034 | 0.0040 | 0.850 | .399 |
| Conduction × WAB-AQ | 0.0033 | 0.0046 | 0.708 | .482 |

Note. Model statistics: *F*(11,56)=1.45, *p*=.178, adjusted R²=.068

Subtype × WAB-AQ interaction effect, *F*(2,56) = 0.36, *p* = .699.

## **Table A.4. Combined Language Network (Reduced Main-Effects Model)**

| Predictor | β | SE | t | p |
| --- | --- | --- | --- | --- |
| Intercept | 0.081 | 0.125 | 0.651 | .518 |
| Broca vs Anomic | 0.048 | 0.047 | 1.022 | .311 |
| Conduction vs Anomic | 0.068 | 0.043 | 1.567 | .123 |
| Broca vs Conduction | −0.021 | 0.044 | −0.473 | .638 |
| WAB-AQ | 0.0015 | 0.00084 | 1.823 | .074 |
| Lesion volume | -1.94×10⁻⁷ | 1.68×10⁻⁷ | -1.161 | .250 |
| Age at stroke | 0.00052 | 0.00117 | 0.443 | .659 |
| Sex (Male) | 0.023 | 0.028 | 0.807 | .423 |
| Days between WAB and rs-fMRI | 0.00001 | 0.00089 | 0.016 | .987 |
| Time post-stroke | 1.68×10⁻⁵ | 8.31×10⁻⁶ | 2.021 | .048* |
| Head motion | -0.024 | 0.021 | -1.119 | .268 |

Note. Model statistics: *F*(9,58)=1.73, *p*=.103, adjusted R²=.089

## **Table A.5. Ventral Stream (Interaction Model)**

| Predictor | β | SE | t | p |
| --- | --- | --- | --- | --- |
| Intercept | 0.589 | 0.597 | 0.987 | .328 |
| Broca vs Anomic | -0.396 | 0.539 | -0.734 | .466 |
| Conduction vs Anomic | -0.440 | 0.594 | -0.742 | .461 |
| Broca vs Conduction | 0.044 | 0.279 | 0.158 | .875 |
| WAB-AQ | -0.0029 | 0.0064 | -0.457 | .649 |
| Lesion volume | -2.76×10⁻⁷ | 2.77×10⁻⁷ | -0.996 | .323 |
| Age at stroke | 0.00033 | 0.00194 | 0.170 | .865 |
| Sex (Male) | -0.008 | 0.047 | -0.169 | .866 |
| Days between WAB and rs-fMRI | -0.00033 | 0.00144 | -0.231 | .818 |
| Time post-stroke | 2.15×10⁻⁵ | 1.33×10⁻⁵ | 1.618 | .111 |
| Head motion | -0.026 | 0.034 | -0.763 | .449 |
| Broca × WAB-AQ | 0.0056 | 0.0064 | 0.884 | .380 |
| Conduction × WAB-AQ | 0.0055 | 0.0073 | 0.752 | .455 |

Note. Model statistics: F(11,56)=1.04, *p*=.423, adjusted R²=.007

Subtype × WAB-AQ interaction effect, *F*(2,56) = 0.39, *p* = .678.

## **Table A.6. Ventral Stream (Reduced Main-Effects Model)**

| Predictor | β | SE | t | p |
| --- | --- | --- | --- | --- |
| Intercept | 0.091 | 0.197 | 0.464 | .644 |
| Broca vs Anomic | 0.077 | 0.073 | 1.047 | .299 |
| Conduction vs Anomic | 0.027 | 0.068 | 0.400 | .691 |
| Broca vs Conduction | 0.049 | 0.068 | 0.721 | .474 |
| WAB-AQ | 0.0026 | 0.0013 | 1.953 | .056 |
| Lesion volume | -2.11×10⁻⁷ | 2.64×10⁻⁷ | -0.801 | .427 |
| Age at stroke | 0.00074 | 0.00185 | 0.399 | .691 |
| Sex (Male) | -0.012 | 0.045 | -0.263 | .794 |
| Days between WAB and rs-fMRI | -0.00048 | 0.00140 | -0.343 | .733 |
| Time post-stroke | 2.11×10⁻⁵ | 1.31×10⁻⁵ | 1.613 | .112 |
| Head motion | -0.026 | 0.034 | -0.784 | .437 |

Note. Model statistics: *F*(9,58)=1.21, *p*=.305, adjusted R²=.028

## **Table A.7. Dorsal Stream (Interaction Model)**

| Predictor | β | SE | t | p |
| --- | --- | --- | --- | --- |
| Intercept | 0.414 | 0.386 | 1.071 | .289 |
| Broca vs Anomic | -0.194 | 0.349 | -0.556 | .580 |
| Conduction vs Anomic | -0.104 | 0.384 | -0.270 | .788 |
| Broca vs Conduction | −0.090 | 0.181 | −0.498 | .620 |
| WAB-AQ | -0.0020 | 0.0041 | -0.480 | .633 |
| Lesion volume | -3.57×10⁻⁷ | 1.79×10⁻⁷ | -1.989 | .052 |
| Age at stroke | -0.00035 | 0.00125 | -0.280 | .781 |
| Sex (Male) | 0.054 | 0.031 | 1.771 | .082 |
| Days between WAB and rs-fMRI | 0.00053 | 0.00093 | 0.571 | .570 |
| Time post-stroke | 1.76×10⁻⁵ | 8.57×10⁻⁶ | 2.047 | .045* |
| Head motion | -0.019 | 0.022 | -0.878 | .384 |
| Broca × WAB-AQ | 0.0026 | 0.0041 | 0.636 | .527 |
| Conduction × WAB-AQ | 0.0023 | 0.0047 | 0.485 | .630 |

Note. Model statistics: *F*(11,56)=1.79, *p*=.079, adjusted R²=.114

Subtype × WAB-AQ interaction effect, *F*(2,56) = 0.21, *p* = .815.

## **Table A.8. Dorsal Stream (Reduced Main-Effects Model)**

| Predictor | β | SE | t | p |
| --- | --- | --- | --- | --- |
| Intercept | 0.184 | 0.127 | 1.450 | .152 |
| Broca vs Anomic | 0.025 | 0.047 | 0.531 | .598 |
| Conduction vs Anomic | 0.096 | 0.044 | 2.163 | .035* |
| Broca vs Conduction | -0.070 | 0.044 | -1.594 | .116 |
| WAB-AQ | 0.00056 | 0.00086 | 0.650 | .518 |
| Lesion volume | -3.28×10⁻⁷ | 1.70×10⁻⁷ | -1.927 | .059 |
| Age at stroke | -0.00015 | 0.00119 | -0.124 | .902 |
| Sex (Male) | 0.051 | 0.029 | 1.790 | .079 |
| Days between WAB and rs-fMRI | 0.00047 | 0.00091 | 0.521 | .604 |
| Time post-stroke | 1.74×10⁻⁵ | 8.44×10⁻⁶ | 2.065 | .043* |
| Head motion | -0.020 | 0.022 | -0.900 | .372 |

Note. Model statistics: *F*(9,58)=2.20, *p*=.035, adjusted R²=.139

**Table A.9. Between-group functional connectivity comparisons: Broca’s vs. Anomic aphasia.**

| **Broca > Anomic** | | | |
| --- | --- | --- | --- |
| ROI1 | ROI2 | Statistic | p-FDR |
| **Left Hemisphere Connections** | | | |
| Superior frontal gyrus-2 Left | Superior temporal sulcus-1 Left | T(70) = 3.33 | 0.036 |
| **Cross-Hemispheric Connections** | | | |
| Superior frontal gyrus-2 Left | Inferior frontal pars opercularis gyrus-1 Right | T(68) = 3.16 | 0.036 |
| Inferior frontal pars opercularis gyrus-1 Right | Superior temporal sulcus-2 Left | T(68) = 3.11 | 0.042 |
| Superior frontal gyrus-2 Left | Middle temporal gyrus-3 Right | T(68) = 2.97 | 0.042 |
| Superior frontal gyrus-2 Left | Superior temporal sulcus-3 Right | T(68) = 2.82 | 0.048 |

Note. Significant results were observed for the Broca’s > Anomic contrast only. No significant connections were identified for the reverse contrast (Anomic > Broca’s).

**Table A.10. Between-group functional connectivity comparisons: Broca’s vs. Conduction aphasia.**

| **Broca > Conduction** | | | |
| --- | --- | --- | --- |
| ROI1 | ROI2 | Statistic | p-FDR |
| **Left Hemisphere Connections** | | | |
| Supramarginal gyrus-7 Left | Middle temporal gyrus-3 Left | T(68) = 3.69 | 0.008 |
| Supramarginal gyrus-7 Left | Superior temporal sulcus-2 Left | T(68) = 3.31 | 0.008 |
| Supramarginal gyrus-7 Left | Superior temporal sulcus-3 Left | T(68) = 3.30 | 0.008 |
| Angular gyrus-2 Left | Superior temporal sulcus-3 Left | T(68) = 3.81 | 0.009 |
| Supramarginal gyrus-7 Left | Superior temporal sulcus-4 Left | T(68) = 2.84 | 0.016 |
| Superior temporal sulcus-4 Left | Inferior frontal pars opercularis gyrus-1 Left | T(68) = 3.29 | 0.018 |
| Superior temporal sulcus-4 Left | Superior temporal sulcus-2 Left | T(68) = 3.22 | 0.018 |
| Superior temporal sulcus-4 Left | Superior temporal gyrus-4 Left | T(68) = 2.84 | 0.023 |
| Supramarginal gyrus-7 Left | Superior temporal gyrus-4 Left | T(68) = 2.51 | 0.028 |
| Supramarginal gyrus-7 Left | Superior temporal sulcus-1 Left | T(68) = 2.51 | 0.028 |
| Superior temporal sulcus-4 Left | Superior temporal sulcus-3 Left | T(68) = 2.59 | 0.036 |
| Supramarginal gyrus-7 Left | Superior frontal gyrus-2 Left | T(68) = 2.38 | 0.036 |
| Superior temporal sulcus-4 Left | Superior temporal sulcus-1 Left | T(68) = 2.46 | 0.039 |
| Middle temporal gyrus-3 Left | Angular gyrus-2 Left | T(68) = 3.12 | 0.041 |
| Superior temporal sulcus-4 Left | Angular gyrus-2 Left | T(68) = 2.41 | 0.041 |
| Superior temporal sulcus-3 Left | Inferior frontal pars opercularis gyrus-1 Left | T(68) = 2.71 | 0.046 |
| **Cross-Hemispheric Connections** | | | |
| Supramarginal gyrus-7 Left | Superior frontal gyrus-2 Right | T(68) = 3.49 | 0.008 |
| Supramarginal gyrus-7 Left | Middle temporal gyrus-3 Right | T(68) = 3.32 | 0.008 |
| Supramarginal gyrus-7 Left | Superior temporal sulcus-2 Right | T(68) = 3.30 | 0.008 |
| Supramarginal gyrus-7 Left | Superior temporal sulcus-4 Right | T(68) = 3.10 | 0.011 |
| Supramarginal gyrus-7 Left | Superior temporal sulcus-1 Right | T(68) = 3.05 | 0.011 |
| Supramarginal gyrus-7 Left | Superior temporal sulcus-3 Right | T(68) = 3.05 | 0.011 |
| Supramarginal gyrus-7 Left | Inferior frontal pars opercularis gyrus-1 Right | T(68) = 3.00 | 0.012 |
| Superior temporal sulcus-4 Left | Superior temporal sulcus-1 Right | T(68) = 3.08 | 0.018 |
| Superior temporal sulcus-4 Left | Superior temporal sulcus-3 Right | T(68) = 3.08 | 0.018 |
| Superior temporal sulcus-4 Left | Superior temporal sulcus-2 Right | T(68) = 3.06 | 0.018 |
| Superior temporal sulcus-4 Left | Middle temporal gyrus-3 Right | T(68) = 3.03 | 0.018 |
| Supramarginal gyrus-7 Left | Inferior frontal pars triangularis gyrus-1 Right | T(68) = 2.68 | 0.023 |
| Supramarginal gyrus-7 Left | Supramarginal gyrus-7 Right | T(68) = 2.66 | 0.023 |
| Superior temporal sulcus-3 Left | Superior temporal sulcus-1 Right | T(68) = 3.11 | 0.028 |
| Supramarginal gyrus-7 Left | Middle temporal gyrus-4 Right | T(68) = 2.52 | 0.028 |
| Superior temporal sulcus-4 Left | Superior temporal sulcus-4 Right | T(68) = 2.68 | 0.032 |
| Superior temporal sulcus-3 Left | Middle temporal gyrus-3 Right | T(68) = 2.94 | 0.035 |
| Middle temporal gyrus-3 Right | Middle temporal gyrus-3 Left | T(68) = 2.92 | 0.037 |
| Superior temporal sulcus-4 Left | Superior temporal gyrus-4 Right | T(68) = 2.55 | 0.037 |
| Superior temporal sulcus-4 Left | Inferior frontal pars opercularis gyrus-1 Right | T(68) = 2.48 | 0.039 |
| Superior temporal sulcus-3 Left | Inferior frontal pars opercularis gyrus-1 Right | T(68) = 2.68 | 0.046 |
| Superior temporal sulcus-3 Left | Angular gyrus-2 Right | T(68) = 2.54 | 0.046 |
| Superior temporal sulcus-3 Left | Superior temporal sulcus-3 Right | T(68) = 2.54 | 0.046 |
| **Conduction > Broca** | | | |
| ROI1 | ROI2 | Statistic | p-FDR |
| **Left Hemisphere Connections** | | | |
| Anterior insula gyrus-2 Left | Superior temporal sulcus-1 Left | T(68) = -3.27 | 0.026 |
| Anterior insula gyrus-2 Left | Precentral sulcus-4 Left | T(68) = -3.37 | 0.026 |
| Anterior insula gyrus-2 Left | Superior temporal sulcus-2 Left | T(68) = -2.62 | 0.037 |
| Anterior insula gyrus-2 Left | Superior frontal gyrus-2 Left | T(68) = -2.64 | 0.037 |
| Anterior insula gyrus-2 Left | Inferior frontal sulcus-2 Left | T(68) = -2.69 | 0.037 |
| Precentral sulcus-4 Left | Anterior insula gyrus-3 Left | T(68) = -3.15 | 0.038 |
| **Cross-Hemispheric Connections** | | | |
| Anterior insula gyrus-2 Left | Superior frontal gyrus-2 Right | T(68) = -3.03 | 0.036 |
| Anterior insula gyrus-2 Left | Precentral sulcus-4 Right | T(68) = -2.72 | 0.037 |
| Anterior insula gyrus-2 Left | Superior temporal sulcus-1 Right | T(68) = -2.72 | 0.037 |
| Anterior insula gyrus-2 Left | Inferior frontal sulcus-2 Right | T(68) = -2.79 | 0.037 |
| Superior temporal sulcus-1 Left | Precentral sulcus-4 Right | T(68) = -3.10 | 0.043 |

**Table A.11. Between-group functional connectivity comparisons: Anomic vs. Conduction aphasia.**

| **Anomic > Conduction** | | | |
| --- | --- | --- | --- |
| ROI1 | ROI2 | Statistic | p-FDR |
| **Left Hemisphere Connections** | | | |
| Precentral sulcus-4 Left | Supramarginal gyrus-7 Left | T(68) = 3.34 | 0.021 |
| **Conduction > Anomic** | | | |
| ROI1 | ROI2 | Statistic | p-FDR |
| **Right Hemisphere Connections** | | | |
| Superior temporal sulcus-2 Right | Superior temporal gyrus-4 Right | T(68) = -4.10 | 0.004 |
| Superior temporal gyrus-4 Right | Superior temporal sulcus-1 Right | T(68) = -3.15 | 0.025 |
| Superior temporal gyrus-4 Right | Superior temporal sulcus-3 Right | T(68) = -3.24 | 0.025 |
| **Left Hemisphere Connections** | | | |
| Anterior insula gyrus-2 Left | Superior frontal gyrus-2 Left | T(68) = -3.31 | 0.010 |
| Anterior insula gyrus-2 Left | Superior temporal sulcus-1 Left | T(68) = -3.43 | 0.010 |
| Superior temporal sulcus-1 Left | Anterior insula gyrus-3 Left | T(68) = -3.33 | 0.011 |
| Superior temporal sulcus-1 Left | Superior frontal gyrus-2 Left | T(68) = -3.05 | 0.013 |
| Anterior insula gyrus-2 Left | Superior temporal sulcus-2 Left | T(68) = -2.99 | 0.015 |
| Anterior insula gyrus-2 Left | Inferior frontal sulcus-2 Left | T(68) = -3.00 | 0.015 |
| Anterior insula gyrus-2 Left | Precentral sulcus-4 Left | T(68) = -2.81 | 0.020 |
| Superior temporal sulcus-1 Left | Inferior frontal pars triangularis gyrus-1 Left | T(68) = -2.73 | 0.023 |
| Superior temporal sulcus-1 Left | Inferior frontal pars opercularis gyrus-1 Left | T(68) = -2.35 | 0.044 |
| **Cross-Hemispheric Connections** | | | |
| Precentral sulcus-4 Right | Inferior frontal pars triangularis gyrus-1 Left | T(68) = -4.21 | 0.002 |
| Precentral sulcus-4 Left | Middle temporal gyrus-4 Right | T(68) = -3.95 | 0.006 |
| Anterior insula gyrus-2 Left | Superior temporal sulcus-2 Right | T(68) = -3.23 | 0.010 |
| Anterior insula gyrus-2 Left | Superior temporal sulcus-3 Right | T(68) = -3.23 | 0.010 |
| Anterior insula gyrus-2 Left | Inferior frontal sulcus-2 Right | T(68) = -3.26 | 0.010 |
| Anterior insula gyrus-2 Left | Superior frontal gyrus-2 Right | T(68) = -3.39 | 0.010 |
| Superior temporal sulcus-1 Left | Inferior frontal pars triangularis gyrus-1 Right | T(68) = -3.13 | 0.011 |
| Superior temporal sulcus-1 Left | Precentral sulcus-4 Right | T(68) = -3.19 | 0.011 |
| Superior temporal sulcus-1 Left | Inferior frontal pars opercularis gyrus-1 Right | T(68) = -3.24 | 0.011 |
| Superior temporal sulcus-1 Left | Middle temporal gyrus-4 Right | T(68) = -3.31 | 0.011 |
| Superior temporal sulcus-1 Left | Anterior insula gyrus-2 Right | T(68) = -3.46 | 0.011 |
| Anterior insula gyrus-2 Left | Superior temporal sulcus-1 Right | T(68) = -2.88 | 0.018 |
| Inferior frontal pars triangularis gyrus-1 Left | Inferior frontal sulcus-2 Right | T(68) = -3.36 | 0.020 |
| Superior temporal sulcus-1 Left | Anterior insula gyrus-3 Right | T(68) = -2.72 | 0.023 |
| Superior temporal sulcus-1 Left | Superior temporal gyrus-4 Right | T(68) = -2.77 | 0.023 |
| Anterior insula gyrus-2 Left | Middle temporal gyrus-3 Right | T(68) = -2.66 | 0.024 |
| Anterior insula gyrus-2 Left | Precentral sulcus-4 Right | T(68) = -2.67 | 0.024 |
| Anterior insula gyrus-2 Left | Inferior frontal pars triangularis gyrus-1 Right | T(68) = -2.69 | 0.024 |
| Inferior frontal pars triangularis gyrus-1 Left | Inferior frontal pars triangularis gyrus-1 Right | T(68) = -3.12 | 0.028 |
| Inferior frontal pars opercularis gyrus-1 Right | Superior temporal sulcus-2 Left | T(68) = -3.30 | 0.028 |
| Inferior frontal pars triangularis gyrus-1 Left | Supramarginal gyrus-7 Right | T(68) = -2.96 | 0.032 |
| Superior frontal gyrus-2 Left | Inferior frontal sulcus-2 Right | T(68) = -2.80 | 0.034 |
| Superior frontal gyrus-2 Left | Inferior frontal pars triangularis gyrus-1 Right | T(68) = -2.80 | 0.034 |
| Superior frontal gyrus-2 Left | Superior frontal gyrus-2 Right | T(68) = -2.84 | 0.034 |
| Superior frontal gyrus-2 Left | Inferior frontal pars opercularis gyrus-1 Right | T(68) = -2.89 | 0.034 |
| Superior temporal sulcus-1 Left | Superior temporal sulcus-1 Right | T(68) = -2.53 | 0.035 |
| Anterior insula gyrus-2 Left | Anterior insula gyrus-2 Right | T(68) = -2.44 | 0.038 |
| Superior temporal sulcus-1 Left | Superior frontal gyrus-2 Right | T(68) = -2.48 | 0.038 |
| Anterior insula gyrus-2 Left | Angular gyrus-2 Right | T(68) = -2.35 | 0.042 |
| Anterior insula gyrus-2 Left | Supramarginal gyrus-7 Right | T(68) = -2.37 | 0.042 |
| Superior temporal sulcus-1 Left | Middle temporal gyrus-3 Right | T(68) = -2.39 | 0.043 |
| Superior frontal gyrus-2 Left | Superior temporal sulcus-1 Right | T(68) = -2.66 | 0.043 |
| Superior frontal gyrus-2 Left | Anterior insula gyrus-2 Right | T(68) = -2.61 | 0.044 |
| Superior temporal sulcus-1 Left | Superior temporal sulcus-2 Right | T(68) = -2.27 | 0.048 |
| Superior temporal sulcus-1 Left | Superior temporal sulcus-3 Right | T(68) = -2.29 | 0.048 |
| Inferior frontal sulcus-2 Right | Anterior insula gyrus-3 Left | T(68) = -2.93 | 0.048 |

**Table A.12. Between-group functional connectivity comparisons: Mild vs. Severe aphasia.**

| **Severe > Mild** | | | |
| --- | --- | --- | --- |
| ROI1 | ROI2 | Statistic | p-FDR |
| **Left Hemisphere Connections** | | | |
| Superior frontal gyrus-2 Left | Anterior insula gyrus-2 Left | T(70) = -4.24 | 0.002 |

Note. Significant results were observed for the Severe > Mild contrast only. No significant connections were identified for the reverse contrast (Mild > Severe).

**Table A.13. Functional connectivity in the All aphasia group.**

| ROI1 | ROI2 | Statistic | p-FDR |
| --- | --- | --- | --- |
| **Right Hemisphere Connections** | | | |
| Superior frontal gyrus-2 Right | Inferior frontal pars opercularis gyrus-1 Right | T(69) = 5.46 | 0.000 |
| Superior frontal gyrus-2 Right | Middle temporal gyrus-3 Right | T(69) = 4.60 | 0.000 |
| Inferior frontal pars opercularis gyrus-1 Right | Inferior frontal pars triangularis gyrus-1 Right | T(69) = 4.52 | 0.000 |
| Precentral sulcus-4 Right | Inferior frontal sulcus-2 Right | T(69) = 4.58 | 0.001 |
| Superior frontal gyrus-2 Right | Anterior insula gyrus-2 Right | T(69) = 4.22 | 0.001 |
| Inferior frontal pars opercularis gyrus-1 Right | Middle temporal gyrus-3 Right | T(69) = 4.22 | 0.001 |
| Inferior frontal pars opercularis gyrus-1 Right | Angular gyrus-2 Right | T(69) = 4.17 | 0.001 |
| Superior frontal gyrus-2 Right | Angular gyrus-2 Right | T(69) = 3.99 | 0.001 |
| Superior temporal sulcus-3 Right | Superior temporal sulcus-4 Right | T(69) = 4.13 | 0.002 |
| Middle temporal gyrus-3 Right | Superior temporal sulcus-3 Right | T(69) = 4.01 | 0.002 |
| Superior frontal gyrus-2 Right | Superior temporal sulcus-1 Right | T(69) = 3.80 | 0.002 |
| Middle temporal gyrus-3 Right | Superior temporal sulcus-2 Right | T(69) = 3.76 | 0.003 |
| Superior temporal sulcus-3 Right | Superior temporal sulcus-1 Right | T(69) = 3.73 | 0.003 |
| Superior temporal sulcus-3 Right | Superior temporal gyrus-4 Right | T(69) = 3.69 | 0.003 |
| Middle temporal gyrus-3 Right | Superior temporal sulcus-1 Right | T(69) = 3.66 | 0.003 |
| Superior temporal sulcus-1 Right | Superior temporal sulcus-2 Right | T(69) = 4.00 | 0.004 |
| Middle temporal gyrus-3 Right | Superior temporal gyrus-4 Right | T(69) = 3.34 | 0.007 |
| Superior frontal gyrus-2 Right | Superior temporal sulcus-3 Right | T(69) = 3.23 | 0.010 |
| Superior temporal sulcus-3 Right | Superior temporal sulcus-2 Right | T(69) = 3.20 | 0.011 |
| Superior frontal gyrus-2 Right | Superior temporal sulcus-2 Right | T(69) = 3.15 | 0.011 |
| Superior temporal sulcus-3 Right | Inferior frontal pars opercularis gyrus-1 Right | T(69) = 3.09 | 0.013 |
| Superior temporal gyrus-4 Right | Superior temporal sulcus-4 Right | T(69) = 3.39 | 0.014 |
| Inferior frontal pars opercularis gyrus-1 Right | Superior temporal sulcus-2 Right | T(69) = 2.84 | 0.023 |
| Inferior frontal pars opercularis gyrus-1 Right | Anterior insula gyrus-2 Right | T(69) = 2.78 | 0.024 |
| Inferior frontal pars opercularis gyrus-1 Right | Superior temporal sulcus-1 Right | T(69) = 2.67 | 0.029 |
| Superior temporal sulcus-1 Right | Superior temporal gyrus-4 Right | T(69) = 2.80 | 0.041 |
| Superior temporal sulcus-1 Right | Superior temporal sulcus-4 Right | T(69) = 2.70 | 0.042 |
| Inferior frontal pars opercularis gyrus-1 Right | Inferior frontal sulcus-2 Right | T(69) = 2.39 | 0.044 |
| Inferior frontal pars opercularis gyrus-1 Right | Superior temporal gyrus-4 Right | T(69) = 2.39 | 0.044 |
| Superior temporal sulcus-3 Right | Inferior frontal pars triangularis gyrus-1 Right | T(69) = 2.56 | 0.049 |
| **Left Hemisphere Connections** | | | |
| Anterior insula gyrus-2 Left | Superior frontal gyrus-2 Left | T(69) = 4.04 | 0.004 |
| Inferior frontal pars triangularis gyrus-1 Left | Inferior frontal pars opercularis gyrus-1 Left | T(69) = 3.97 | 0.005 |
| Superior frontal gyrus-2 Left | Inferior frontal pars opercularis gyrus-1 Left | T(69) = 3.24 | 0.023 |
| **Cross-Hemispheric Connections** | | | |
| Inferior frontal pars opercularis gyrus-1 Right | Superior frontal gyrus-2 Left | T(69) = 3.17 | 0.014 |
| Inferior frontal pars opercularis gyrus-1 Right | Inferior frontal pars triangularis gyrus-1 Left | T(69) = 2.91 | 0.022 |
| Superior frontal gyrus-2 Right | Superior frontal gyrus-2 Left | T(69) = 2.66 | 0.037 |
| Superior frontal gyrus-2 Right | Anterior insula gyrus-2 Left | T(69) = 2.62 | 0.037 |
| Inferior frontal pars opercularis gyrus-1 Right | Inferior frontal pars opercularis gyrus-1 Left | T(69) = 2.54 | 0.038 |
| Inferior frontal pars opercularis gyrus-1 Right | Anterior insula gyrus-2 Left | T(69) = 2.46 | 0.043 |
| Angular gyrus-2 Right | Superior frontal gyrus-2 Left | T(69) = 2.84 | 0.044 |
| Angular gyrus-2 Right | Inferior frontal pars opercularis gyrus-1 Left | T(69) = 2.81 | 0.044 |
| Angular gyrus-2 Right | Supramarginal gyrus-7 Left | T(69) = -2.78 | 0.044 |
| Superior frontal gyrus-2 Left | Anterior insula gyrus-2 Right | T(69) = 2.75 | 0.047 |

**Table A.14. Functional connectivity in the All aphasia group associated with aphasia severity.**

| ROI1 | ROI2 | Statistic | p-FDR |
| --- | --- | --- | --- |
| **Right Hemisphere Connections** | | | |
| Superior temporal sulcus-4 Right | Angular gyrus-2 Right | T(69) = 2.82 | 0.048 |
| Superior temporal sulcus-4 Right | Superior frontal gyrus-2 Right | T(69) = 2.56 | 0.050 |
| Superior temporal sulcus-4 Right | Anterior insula gyrus-2 Right | T(69) = 2.51 | 0.050 |
| **Cross-Hemispheric Connections** | | | |
| Superior temporal sulcus-4 Right | Middle temporal gyrus-3 Left | T(69) = 3.81 | 0.009 |
| Superior temporal sulcus-4 Right | Superior temporal sulcus-3 Left | T(69) = 3.37 | 0.019 |
| Superior temporal sulcus-3 Left | Inferior frontal pars triangularis gyrus-1 Right | T(69) = 3.20 | 0.032 |
| Superior temporal sulcus-4 Right | Superior temporal gyrus-4 Left | T(69) = 3.02 | 0.036 |
| Middle temporal gyrus-3 Left | Superior temporal sulcus-3 Right | T(69) = 3.15 | 0.038 |
| Middle temporal gyrus-3 Left | Superior temporal sulcus-2 Right | T(69) = 2.94 | 0.042 |
| Middle temporal gyrus-3 Left | Middle temporal gyrus-3 Right | T(69) = 2.87 | 0.042 |
| Middle temporal gyrus-3 Left | Inferior frontal pars triangularis gyrus-1 Right | T(69) = 2.74 | 0.048 |
| Middle temporal gyrus-3 Left | Superior temporal gyrus-4 Right | T(69) = 2.67 | 0.049 |
| Superior temporal sulcus-4 Right | Superior temporal sulcus-1 Left | T(69) = 2.63 | 0.050 |
| Superior temporal sulcus-4 Right | Superior frontal gyrus-2 Left | T(69) = 2.53 | 0.050 |
| Superior temporal sulcus-4 Right | Anterior insula gyrus-3 Left | T(69) = -2.53 | 0.050 |

**Table A.15. Functional connectivity in the Anomic aphasia group.**

| ROI1 | ROI2 | Statistic | p-FDR |
| --- | --- | --- | --- |
| **Left Hemisphere Connections** | | | |
| Supramarginal gyrus-7 Left | Middle temporal gyrus-4 Left | T(13) = -4.89 | 0.015 |

**Table A.16. Functional connectivity in the Anomic aphasia group associated with aphasia severity.**

| ROI1 | ROI2 | Statistic | p-FDR |
| --- | --- | --- | --- |
| **Left Hemisphere Connections** | | | |
| Supramarginal gyrus-7 Left | Middle temporal gyrus-4 Left | T(13) = 4.64 | 0.022 |

**Table A.17. Functional connectivity in the Broca aphasia group.**

| ROI1 | ROI2 | Statistic | p-FDR |
| --- | --- | --- | --- |
| **Right Hemisphere Connections** | | | |
| Superior temporal gyrus-4 Right | Middle temporal gyrus-3 Right | T(32) = 4.34 | 0.004 |
| Superior frontal gyrus-2 Right | Inferior frontal pars opercularis gyrus-1 Right | T(32) = 4.17 | 0.007 |
| Superior temporal sulcus-3 Right | Superior temporal sulcus-4 Right | T(32) = 4.09 | 0.007 |
| Superior temporal sulcus-3 Right | Superior temporal gyrus-4 Right | T(32) = 3.77 | 0.007 |
| Superior temporal sulcus-3 Right | Superior temporal sulcus-1 Right | T(32) = 3.75 | 0.007 |
| Superior temporal sulcus-4 Right | Superior temporal sulcus-1 Right | T(32) = 3.77 | 0.008 |
| Superior temporal sulcus-4 Right | Superior temporal sulcus-2 Right | T(32) = 3.70 | 0.008 |
| Superior temporal sulcus-2 Right | Precentral sulcus-4 Right | T(32) = 3.67 | 0.013 |
| Superior temporal sulcus-1 Right | Superior temporal gyrus-4 Right | T(32) = 3.53 | 0.013 |
| Superior temporal gyrus-4 Right | Superior frontal gyrus-2 Right | T(32) = 3.39 | 0.014 |
| Superior temporal sulcus-3 Right | Superior frontal gyrus-2 Right | T(32) = 3.33 | 0.016 |
| Superior temporal sulcus-3 Right | Superior temporal sulcus-2 Right | T(32) = 3.20 | 0.016 |
| Superior temporal sulcus-3 Right | Middle temporal gyrus-3 Right | T(32) = 3.19 | 0.016 |
| Superior temporal gyrus-4 Right | Superior temporal sulcus-2 Right | T(32) = 3.25 | 0.017 |
| Superior temporal sulcus-3 Right | Precentral sulcus-4 Right | T(32) = 3.13 | 0.017 |
| Inferior frontal pars opercularis gyrus-1 Right | Inferior frontal pars triangularis gyrus-1 Right | T(32) = 3.56 | 0.018 |
| Precentral sulcus-4 Right | Inferior frontal sulcus-2 Right | T(32) = 3.37 | 0.025 |
| Superior temporal sulcus-1 Right | Middle temporal gyrus-3 Right | T(32) = 3.18 | 0.025 |
| Precentral sulcus-4 Right | Inferior frontal pars triangularis gyrus-1 Right | T(32) = 3.10 | 0.025 |
| Precentral sulcus-4 Right | Superior temporal sulcus-4 Right | T(32) = 3.10 | 0.025 |
| Superior temporal sulcus-4 Right | Middle temporal gyrus-3 Right | T(32) = 3.03 | 0.028 |
| Superior temporal sulcus-4 Right | Superior temporal gyrus-4 Right | T(32) = 2.98 | 0.028 |
| Precentral sulcus-4 Right | Supramarginal gyrus-7 Right | T(32) = 2.97 | 0.029 |
| Superior temporal sulcus-2 Right | Superior temporal sulcus-1 Right | T(32) = 2.98 | 0.034 |
| Precentral sulcus-4 Right | Superior temporal sulcus-1 Right | T(32) = 2.84 | 0.034 |
| Superior temporal gyrus-4 Right | Inferior frontal pars triangularis gyrus-1 Right | T(32) = 2.73 | 0.046 |

**Table A.18. Functional connectivity in the Broca aphasia group associated with aphasia severity.**

| ROI1 | ROI2 | Statistic | p-FDR |
| --- | --- | --- | --- |
| **Left Hemisphere Connections** | | | |
| Superior frontal gyrus-2 Left | Middle temporal gyrus-3 Left | T(32) = 4.65 | 0.002 |
| Middle temporal gyrus-3 Left | Inferior frontal pars opercularis gyrus-1 Left | T(32) = 3.25 | 0.014 |
| Superior temporal sulcus-1 Left | Superior temporal sulcus-4 Left | T(32) = 3.26 | 0.016 |
| Superior temporal sulcus-1 Left | Superior frontal gyrus-2 Left | T(32) = 3.15 | 0.016 |
| Superior temporal sulcus-4 Left | Inferior frontal pars opercularis gyrus-1 Left | T(32) = 3.15 | 0.022 |
| Superior temporal sulcus-4 Left | Superior frontal gyrus-2 Left | T(32) = 2.81 | 0.037 |
| **Cross-Hemispheric Connections** | | | |
| Middle temporal gyrus-3 Left | Inferior frontal pars opercularis gyrus-1 Right | T(32) = 4.22 | 0.003 |
| Middle temporal gyrus-3 Left | Middle temporal gyrus-3 Right | T(32) = 3.77 | 0.007 |
| Superior temporal sulcus-1 Left | Superior temporal sulcus-2 Right | T(32) = 3.87 | 0.008 |
| Superior temporal sulcus-1 Left | Superior frontal gyrus-2 Right | T(32) = 3.85 | 0.008 |
| Middle temporal gyrus-3 Left | Superior frontal gyrus-2 Right | T(32) = 3.30 | 0.014 |
| Middle temporal gyrus-3 Left | Superior temporal sulcus-4 Right | T(32) = 3.26 | 0.014 |
| Superior temporal sulcus-1 Left | Middle temporal gyrus-3 Right | T(32) = 3.47 | 0.015 |
| Superior temporal sulcus-1 Left | Inferior frontal pars opercularis gyrus-1 Right | T(32) = 3.35 | 0.016 |
| Superior temporal sulcus-1 Left | Superior temporal sulcus-4 Right | T(32) = 3.13 | 0.016 |
| Superior temporal sulcus-4 Left | Middle temporal gyrus-3 Right | T(32) = 3.32 | 0.022 |
| Superior temporal sulcus-4 Left | Superior temporal sulcus-2 Right | T(32) = 3.21 | 0.022 |
| Superior temporal sulcus-4 Left | Superior frontal gyrus-2 Right | T(32) = 3.19 | 0.022 |
| Middle temporal gyrus-3 Left | Superior temporal sulcus-3 Right | T(32) = 3.00 | 0.023 |
| Middle temporal gyrus-3 Left | Superior temporal sulcus-2 Right | T(32) = 2.91 | 0.025 |
| Superior temporal sulcus-1 Left | Inferior frontal pars triangularis gyrus-1 Right | T(32) = 2.79 | 0.034 |
| Inferior frontal pars opercularis gyrus-1 Right | Superior temporal sulcus-3 Left | T(32) = 3.15 | 0.037 |
| Superior temporal sulcus-4 Left | Inferior frontal pars opercularis gyrus-1 Right | T(32) = 2.87 | 0.037 |
| Middle temporal gyrus-3 Left | Superior temporal gyrus-4 Right | T(32) = 2.63 | 0.045 |

**Table A.19. Functional connectivity in the Mild aphasia group.**

| ROI1 | ROI2 | Statistic | p-FDR |
| --- | --- | --- | --- |
| **Cross-Hemispheric Connections** | | | |
| Superior frontal gyrus-2 Left | Middle temporal gyrus-3 Right | T(17) = 4.77 | 0.006 |
| Superior frontal gyrus-2 Left | Superior temporal sulcus-2 Right | T(17) = 4.41 | 0.006 |
| Superior frontal gyrus-2 Left | Angular gyrus-2 Right | T(17) = 3.99 | 0.010 |
| Anterior insula gyrus-2 Right | Superior temporal sulcus-2 Left | T(17) = 4.01 | 0.028 |
| Inferior frontal pars triangularis gyrus-1 Right | Anterior insula gyrus-3 Left | T(17) = 3.81 | 0.044 |

**Table A.20. Functional connectivity in the Mild aphasia group associated with aphasia severity.**

| ROI1 | ROI2 | Statistic | p-FDR |
| --- | --- | --- | --- |
| **Cross-Hemispheric Connections** | | | |
| Superior frontal gyrus-2 Left | Middle temporal gyrus-3 Right | T(17) = -3.55 | 0.038 |
| Superior frontal gyrus-2 Left | Superior temporal sulcus-2 Right | T(17) = -3.63 | 0.038 |

**Table A.21. Functional connectivity in the Severe aphasia group.**

| ROI1 | ROI2 | Statistic | p-FDR |
| --- | --- | --- | --- |
| **Right Hemisphere Connections** | | | |
| Superior temporal sulcus-3 Right | Superior temporal gyrus-4 Right | T(19) = 3.43 | 0.029 |
| Middle temporal gyrus-3 Right | Superior temporal gyrus-4 Right | T(19) = 3.32 | 0.038 |
| Superior temporal sulcus-4 Right | Superior temporal sulcus-1 Right | T(19) = 3.20 | 0.049 |
| Superior temporal sulcus-4 Right | Superior temporal sulcus-3 Right | T(19) = 3.06 | 0.049 |
| Superior temporal sulcus-4 Right | Inferior frontal pars triangularis gyrus-1 Right | T(19) = 2.88 | 0.049 |
| **Cross-Hemispheric Connections** | | | |
| Superior temporal sulcus-3 Left | Superior temporal sulcus-3 Right | T(19) = -3.73 | 0.022 |
| Superior temporal sulcus-3 Left | Superior temporal sulcus-4 Right | T(19) = -4.03 | 0.022 |
| Superior temporal sulcus-3 Left | Middle temporal gyrus-3 Right | T(19) = -3.46 | 0.027 |
| Superior temporal sulcus-3 Right | Middle temporal gyrus-3 Left | T(19) = -3.52 | 0.029 |
| Middle temporal gyrus-3 Right | Middle temporal gyrus-3 Left | T(19) = -3.32 | 0.038 |
| Middle temporal gyrus-3 Left | Inferior frontal pars triangularis gyrus-1 Right | T(19) = -3.26 | 0.042 |
| Superior temporal sulcus-3 Left | Inferior frontal pars triangularis gyrus-1 Right | T(19) = -3.09 | 0.046 |
| Superior temporal sulcus-4 Right | Middle temporal gyrus-3 Left | T(19) = -2.91 | 0.049 |
| Superior temporal sulcus-4 Right | Superior temporal sulcus-1 Left | T(19) = -3.18 | 0.049 |

**Table A.22. Functional connectivity associated with aphasia severity in the Severe aphasia group.**

| ROI1 | ROI2 | Statistic | p-FDR |
| --- | --- | --- | --- |
| **Left Hemisphere Connections** | | | |
| Superior temporal sulcus-4 Left | Angular gyrus-2 Left | T(19) = -3.95 | 0.027 |
